# Influenza A Virus-Induced circRNA circMerTK Negatively Regulates Innate Antiviral Responses

**DOI:** 10.1128/spectrum.03637-22

**Published:** 2023-02-27

**Authors:** Haori Qiu, Bincai Yang, Yuhai Chen, Qianwen Zhu, Faxin Wen, Min Peng, Guoqing Wang, Guijie Guo, Biao Chen, Mohamed Maarouf, Min Fang, Ji-Long Chen

**Affiliations:** a Fujian Agriculture and Forestry University, Fuzhou, China; b Key Laboratory of Animal Pathogen Infection and Immunology of Fujian Province, College of Animal Sciences, Fujian Agriculture and Forestry University, Fuzhou, China; c CAS Key Laboratory of Pathogenic Microbiology and Immunology, Institute of Microbiology, Chinese Academy of Sciences (CAS), Beijing, China; d Department of Virology, Faculty of Veterinary Medicine, Suez Canal University, Egypt; David Geffen School of Medicine at UCLA

**Keywords:** influenza A virus, circular RNA, innate immune response, interferon-stimulating genes, MerTK

## Abstract

Circular RNAs (circRNAs) are an important subclass of noncoding RNAs implicated in the regulation of multiple biological processes. However, the functional involvement of circRNAs in the pathogenesis of influenza A viruses (IAVs) remains largely unknown. Here, we employed RNA sequencing (RNA-Seq) to examine the differentially expressed circRNAs in mouse lung tissues challenged or not challenged with IAV to evaluate the impact of viral infection on circRNAs *in vivo*. We observed that 413 circRNAs exhibited significantly altered levels following IAV infection. Among these, circMerTK, the derivative of myeloid-epithelial-reproductive tyrosine kinase (MerTK) pre-mRNA, was highly induced by IAV. Interestingly, circMerTK expression was also increased upon infection with multiple DNA and RNA viruses in human and animal cell lines, and thus it was selected for further studies. Poly(I:C) and interferon β (IFN-β) stimulated circMerTK expression, while RIG-I knockout and IFNAR1 knockout cell lines failed to elevate circMerTK levels after IAV infection, demonstrating that circMerTK is regulated by IFN signaling. Furthermore, circMerTK overexpression or silencing accelerated or impeded IAV and Sendai virus replication, respectively. Silencing circMerTK enhanced the production of type I IFNs and interferon-stimulating genes (ISGs), whereas circMerTK overexpression suppressed their expression at both the mRNA and protein levels. Notably, altering circMerTK expression had no effect on the MerTK mRNA level in cells infected or not infected with IAV, and vice versa. In addition, human circMerTK and mouse homologs functioned similarly in antiviral responses. Together, these results identify circMerTK as an enhancer of IAV replication through suppression of antiviral immunity.

**IMPORTANCE** CircRNAs are an important class of noncoding RNAs characterized by a covalently closed circular structure. CircRNAs have been proven to impact numerous cellular processes, where they conduct specialized biological activities. In addition, circRNAs are believed to play a crucial role in regulating immune responses. Nevertheless, the functions of circRNAs in the innate immunity against IAV infection remain obscure. In this study, we employed transcriptomic analysis to investigate the alterations in circRNAs expression following IAV infection *in vivo*. It was found that expression of 413 circRNAs was significantly altered, of which 171 were upregulated, and 242 were downregulated following the IAV infection. Interestingly, circMerTK was identified as a positive regulator of IAV replication in both human and mouse hosts. CircMerTK was shown to influence IFN-β production and its downstream signaling, enhancing IAV replication. This finding provides new insights into the critical roles of circRNAs in regulating antiviral immunity.

## INTRODUCTION

Influenza/flu caused by influenza A viruses (IAVs) is an infectious zoonotic respiratory illness. IAVs, members of the *Orthomyxoviridae* family, are notorious for causing seasonal epidemics and occasional pandemics. According to the World Health Organization, influenza A and B viruses cause 3 to 5 million severe acute respiratory illnesses and 290,000 to 650,000 deaths annually (1). There is a pressing need to better understand the interaction between influenza viruses and their hosts and the precise mechanisms underlying influenza virus pathogenesis. To this end, it is critical to determine the key molecules modulating the immune response to influenza virus infection, since these molecules might serve as primary drug targets or diagnostic biomarkers.

Circular RNAs (circRNAs) are an important subclass of noncoding RNAs which are implicated in the regulation of multiple biological processes. CircRNAs lack both the 5′-terminal cap and 3′-terminal poly(A) tail structures. They are mainly generated by back-splicing of their parental pre-mRNA (2, 3). As a result, they exhibit far greater resistance to exoribonuclease degradation than linear RNAs (4). Despite these well-defined characteristics, the functions of most circRNAs remain unidentified. Nevertheless, numerous cellular and viral circRNAs have been shown to play crucial roles in regulating gene expression and protein activity (5, 6). Emerging shreds of evidence suggest that circRNAs can conduct their functions via a wide range of mechanisms, including but not limited to microRNA sponging, regulation of gene expression via modulating transcription and splicing, and interaction with RNA-binding proteins. Although the majority of circRNAs are thought to be noncoding, some circRNAs may function by undergoing translation under specific conditions (7).

Recently, an increasing number of studies have suggested the involvement of various circRNAs in regulating or modulating viral infection and antiviral innate immunity. For instance, some circRNAs are reported to compete with the viral RNA for binding with NF90/NF110, a dsRNA-binding protein, and therefore play a role in innate antiviral immunity. In non-infection settings, some circRNAs associate with NF90/NF110 to form circRNA-protein complexes (circRNPs), preventing NF90/NF110 from activating a nonspecific immune response. Upon viral infection, NF90/NF110 is exported to the cytoplasm, where it dissociates from circRNPs to facilitate its binding to viral mRNAs and enhance antiviral immunity. Hence, it has been suggested that these circRNAs may act as a molecular reservoir for NF90/NF110 that could trigger a rapid immune response (8). Some other circRNAs have been investigated in specific viral infection scenarios, such as circTNFAIP3, which has been demonstrated to promote deltacoronavirus replication by inhibiting apoptosis (9). In addition, it has been shown that Hantaan virus (HTNV), which causes hemorrhagic fever with renal syndrome, can alter the expression of multiple circRNAs. For example, HTNV can induce the production of circ_0000479, which appeared to sponge to miR-149-5p, leading to elevated RIG-I levels and impeding viral replication (10).

Importantly, interactions between circRNAs and influenza virus have also been addressed in a few studies. For instance, circ-GATAD2A was reported to boost influenza virus replication via suppressing VPS34-dependent autophagy *in vitro*, which restricts influenza virus infection (11). Moreover, circRNA_0050463 was found to bind with miR-33b-5p, hence promoting eukaryotic translation elongation factor 1 alpha 1 expression and enhancing IAV replication (12). Additionally, the circRNA AIVR has been shown to impede IAV replication primarily by absorbing miR-330-3p, preventing it from binding to CREB binding protein mRNA, a necessary component for interferon (IFN) production (13). Despite the progress that has been made in understanding the relationship between influenza virus infection and circRNAs, the functional involvement of these RNAs in IAV pathogenesis and their contribution to antiviral immunity remain to be further determined.

Myeloid-epithelial-reproductive tyrosine kinase (MerTK) is a member of the Tyro3-Axl-MerTK (TAM) family that is commonly overexpressed in a variety of human cancers and been proven to suppress anti-tumor immunity (14, 15). Furthermore, it is thought that MerTK is an antiviral innate immunity factor that synergistically dampens innate immune responses, favoring virus propagation, and it has been implicated in innate tolerance regulation after viral infection (16, 17). It has been revealed that TAM-deficient dendritic cells are less susceptible to infection by enveloped viruses. This was due to a substantial increase in type I IFN production (18). MerTK has also been implicated in the pathogenesis of two members of the Flaviviridae, classical swine fever virus (CSFV) and bovine viral diarrhea virus (BVDV), by inhibiting innate immune responses, and facilitating CSFV entry, resulting in accelerated viral replication (16). MerTK was also found to mediate immunosuppressive mechanisms during vesicular stomatitis virus (VSV) infection and consequently restrict antiviral responses. MerTK knockout mice exhibited significantly increased production of IFN-α and key interferon-stimulating genes (ISGs) after VSV infection (17). CircMerTK, on the other hand, is a circular RNA derived from MerTK pre-mRNA, and has been demonstrated to be significantly downregulated in hepatocellular carcinoma (HCC) tissues and identified as a potential new diagnostic biomarker for HCC (19). Nevertheless, the involvement of circMerTK in the innate antiviral immunity against viral infection is still elusive.

In this study, we identified circMerTK as a key regulator of innate immune responses to IAV infection in human and mouse cells. Both *in vivo* and *in vitro* experiments showed that circMerTK was significantly induced by infection with IAV and several other viruses. These experiments further established the importance of circMerTK in the pathogenesis of IAV. CircMerTK was found to be a promoter for IAV replication by suppressing IFN-β production and its downstream signaling.

## RESULTS

### CircMerTK is identified as an IAV-induced circRNA *in vivo*.

**FIG 1:**
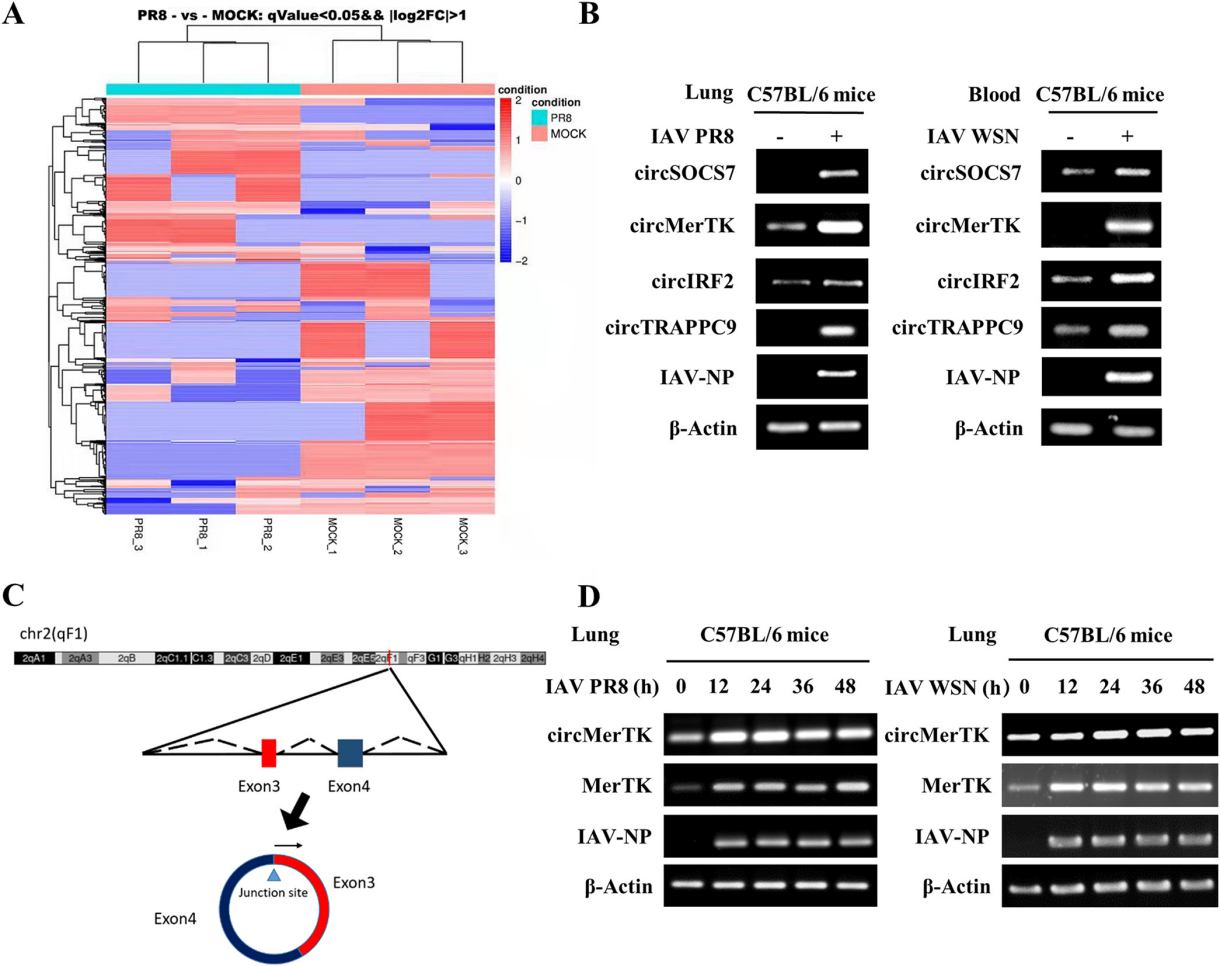
circMerTK is induced by influenza A virus (IAV) infection *in vivo*. (A) Ten C57BL/6 mice were divided into two groups and inoculated with phosphate-buffered saline (PBS) or IAV PR8, and lung samples were collected at 2 days postinfection (dpi) for RNA sequencing (RNA-seq) analysis. Differentially expressed circRNAs are shown in the heatmap. (B) The expression of the four selected circRNAs was confirmed by reverse transcription-PCR (RT-PCR) using divergent primers in the lungs and blood samples of IAV PR8- and IAV WSN-challenged mice. (C) Schematic diagram of circMerTK formed by back-splicing from the mouse MerTK gene at chromosome 2. (D) RT-PCR was used to determine circMerTK, MerTK mRNA, and IAV-NP RNA levels in the lungs of mice challenged with IAV PR8 or IAV WSN for the indicated time points. Representative data from three independent experiments are shown. IAV-NP, IAV nucleoprotein; MerTK, myeloid-epithelial-reproductive tyrosine kinase.

To identify functional circRNAs involved in IAV pathogenesis, we employed RNA sequencing (RNA-seq) to analyze circRNA transcriptomes in the lungs of C57BL/6 mice infected with IAV PR8 virus or treated with phosphate-buffered saline (PBS). Then, log_2_-fold changes in circRNAs were calculated and clustered based on RNA-Seq analysis of the transcriptome, and a heatmap was constructed (Fig. 1A). These experiments identified 5,158 distinct circRNAs. Among these, 413 exhibited significantly changed expression following virus infection, with *P* < 0.05, including 242 circRNAs that were downregulated and 171 circRNAs that showed increased expression.

We then opted to further examine four circRNAs, circSOCS7, circMerTK, circIRF2, and circTRAPPC9, because they exhibited substantially elevated expression following viral infection and are hosted by essential genes (16, 20–22). The expression of these circRNAs in mouse lungs was subjected to reverse transcription-PCR (RT-PCR) to confirm the transcriptome data from RNA-Seq using specific primers that can exclusively target circRNAs. Moreover, the selected circRNAs were also examined in blood samples from C57BL/6 mice challenged with IAV WSN for 48 h. The results showed that all four selected circRNAs were upregulated by IAV PR8 and IAV WSN infection (Fig. 1B, Fig. S1A in the supplemental material). We then decided to focus principally on studying circMerTK because its expression was highly induced by viral infection *in vivo* and it is derived from the pre-mRNA of MerTK, a crucial gene that has been previously implicated in innate immunity (16, 17, 23). We identified circMerTK as a 275-base circRNA by cloning and sequencing. It is an exonic circRNA generated by back-splicing exons 3 and 4 of the MerTK pre-mRNA (Fig. 1C, Fig. S1B and C). Then, a time-course study was performed to determine the dynamic expression of circMerTK during IAV infection. We observed that circMerTK and MerTK mRNA were consistently induced in the lungs of C57BL/6 mice infected with IAV PR8 or WSN strain (Fig. 1D). Together, these experiments demonstrate that circMerTK is a novel circular RNA that is upregulated following infection with IAV strains *in vivo*, suggesting that it may be involved in the virus-host interaction.

### CircMerTK is highly expressed in a wide variety of human and animal cell lines following PR8 infection.

**FIG 2:**
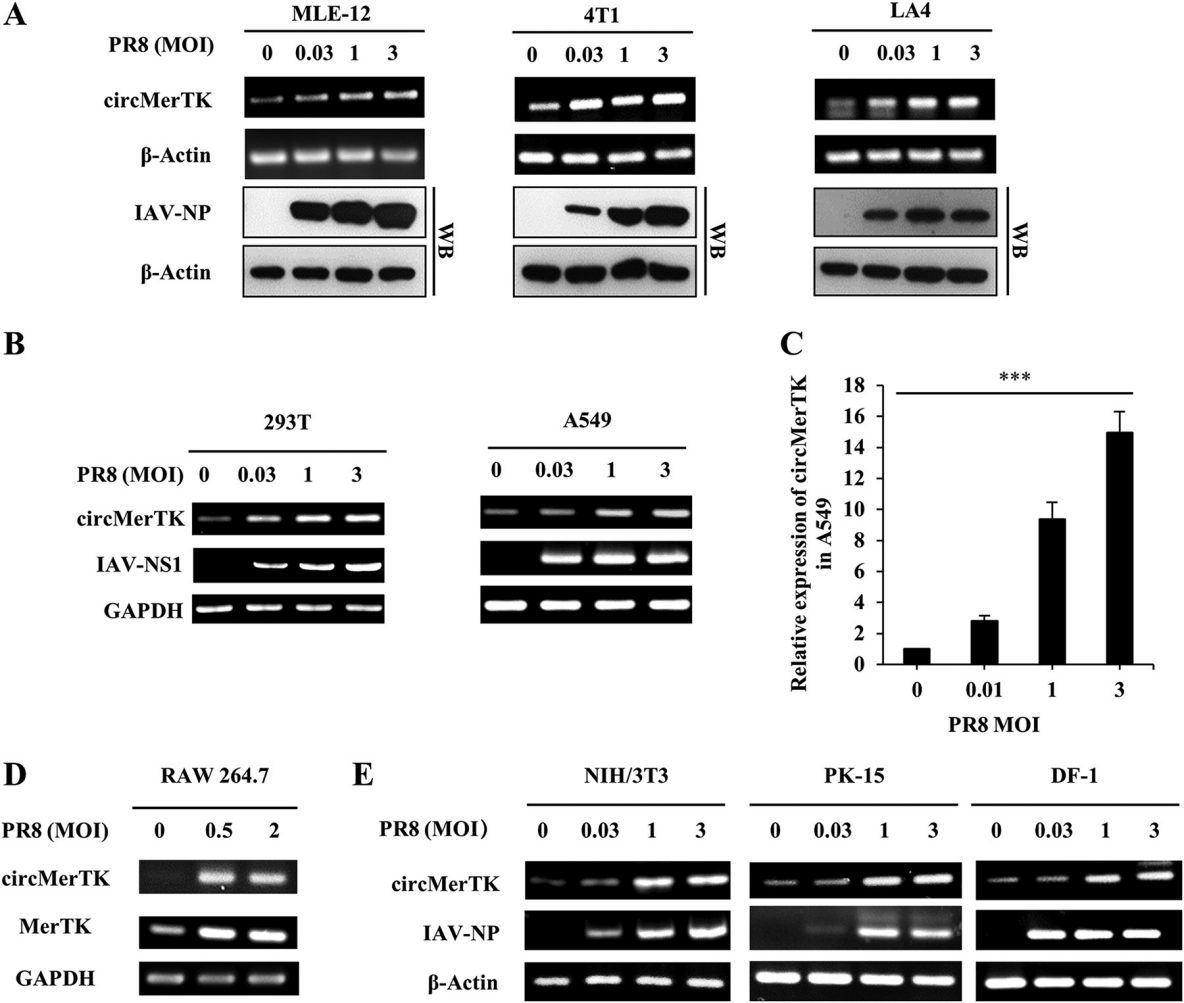
circMerTK is highly expressed in a wide variety of human and animal cells following IAV PR8 infection. (A) Expression of circMerTK was examined by RT-PCR in indicated cells infected with IAV PR8 at the indicated multiplicities of infection (MOIs) for 14 h. The IAV-NP level was determined by Western blotting. (B) Expression of circMerTK was examined by RT-PCR in 293T and A549 cell lines after infection with IAV PR8 at the indicated MOIs for 14 h. (C) Reverse transcription-quantitative PCR (RT-qPCR) was used to evaluate circMerTK expression levels in A549 cells after challenge with IAV PR8 at the indicated MOIs for 14 h. (D) Expression of circMerTK and linear MerTK mRNA was examined by RT-PCR in RAW 264.7 cell line after infection with IAV PR8 at the indicated MOIs for 14 h. (E) NIH/3T3, PK-15, and DF-1 cell lines were infected with IAV PR8 for 14 h and the circMerTK RNA level was determined by RT-PCR. Representative data from three independent experiments are shown. Data represent the mean values ± standard deviation (SD; *n* = 3; ***, *P* < 0.001; *P* value calculated by one-way analysis of variance [ANOVA]). IAV-NS1, IAV nonstructural protein 1.

Next, we tested whether circMerTK could be induced *in vitro* by viral infection. To address this, we conducted *in vitro* experiments to examine the expression patterns in human and mouse cell lines infected with IAV. The BLAST result showed that the nucleic acid sequence homology was 86% between the human circMerTK and mouse circMerTK (Fig. S1D). Using the IAV PR8 strain, we investigated the impacts of different viral multiplicities of infection (MOIs) on circMerTK expression in several mouse cell lines (murine lung epithelial-12 cells [MLE-12], mouse breast cancer cells [4T1], and mouse lung adenoma epithelial-4 cells [LA4]). Notably, circMerTK expression levels in MLE-12, 4T1, and LA4 cell lines were correlated with viral MOIs, i.e., a MOI of 3 induced the highest circMerTK expression level (Fig. 2A). On the other hand, we examined the expression of circMerTK in human cell lines (human embryonic kidney cells [293T] and human lung epithelial cells [A549]) after challenge with IAV PR8 for 14 h. Similarly, circMerTK expression levels were significantly elevated in 293T and A549 cell lines following viral infection (Fig. 2B). These observations were further confirmed in the A549 cells by reverse transcription-quantitative PCR (RT-qPCR) (Fig. 2C). Additionally, we measured the expression of circMerTK and MerTK mRNA in RAW 264.7, a murine macrophage cell line, after challenging it with IAV PR8. Both circMerTK and MerTK mRNA appeared to be upregulated in response to IAV PR8 infection (Fig. 2D).

Analysis of circular RNAs databases indicated that circMerTK was conserved in a wide range of species. Thus, we asked whether other unreported species could also express circMerTK, and whether its expression in other species could be upregulated in response to viral infection. To test this possibility, we blasted the linear sequences of circMerTK in GenBank. The circMerTK homologs were identified in Gallus gallus (chicken) and Sus scrofa (pig) transcriptomes. Next, special divergent primers were designed to amplify the back-splicing junction sites (Fig. S1A). NIH/3T3 mouse embryonic fibroblast cell line, PK-15 pig kidney cell line, and DF-1 chicken fibroblast cell line were challenged with IAV PR8 at various MOIs. Indeed, a specific band was amplified and validated by sequencing for each of these species, demonstrating that circMerTK is induced by IAV infection in mouse, pig, and chicken cells (Fig. 2E). These results reveal that circMerTK is upregulated following infection with IAV in a wide variety of human and animal cell lines, implying a potentially conserved role in the response to viral infection.

### Various RNA and DNA viruses can trigger the upregulation of circMerTK.

**FIG 3:**
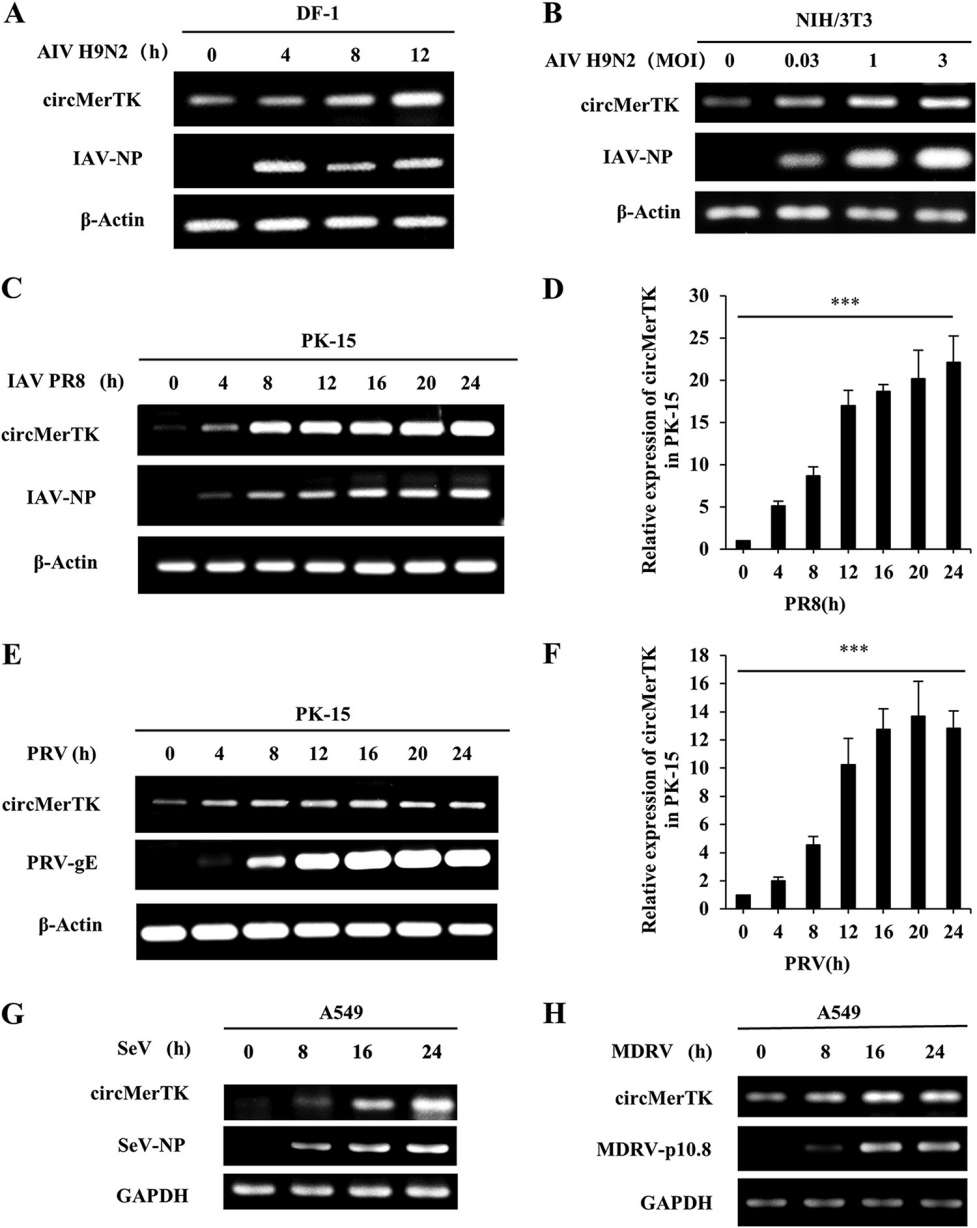
Various DNA and RNA viruses can trigger the upregulation of circMerTK. (A) Expression of circMerTK was examined by RT-PCR in DF1 cell line infected with AIV H9N2 at indicated time points. (B) Expression of circMerTK was examined by RT-PCR in NIH/3T3 cell line infected with AIV H9N2 at the indicated MOIs for 14 h. (C and D) PK-15 cell line was infected with IAV PR8 at indicated time points and the expression level of circMerTK was examined by (C) RT-PCR and (D) RT-qPCR. (E and F) PK-15 cell line was infected with PRV at indicated time points, and the expression level of circMerTK was examined by (E) RT-PCR and (F) RT-qPCR. (G) A549 cell line was infected with SeV or (H) MDRV at indicated time points, and the expression level of circMerTK was examined by RT-PCR. Representative data from three independent experiments are shown. Data represent the mean values ± SD (*n* = 3; ***, *P* < 0.001; *P* value calculated by one-way ANOVA). AIV, avian influenza virus; PRV, pseudorabies virus; SeV, Sendai virus; MDRV, Muscovy duck reovirus; PRV-gE, PRV envelope glycoprotein; SeV-NP, SeV nucleocapsid protein; MDRV-p10.8, MDRV p10.8 protein.

Next, we asked whether circMerTK was induced only by the IAV PR8 and WSN strains, or whether other IAV subtypes and other RNA or DNA viruses could trigger its upregulation. To this end, we challenged the DF-1 cell line with H9N2 avian influenza virus (AIV, negative-stranded ssRNA genome) over different time points. Clearly, circMerTK expression increased as the infection progressed, demonstrating a substantial linkage between H9N2 virus infection and circMerTK expression (Fig. 3A). We also tested the NIH/3T3 cell line, infecting it with AIV H9N2 at various MOIs. CircMerTK expression was enhanced with increasing MOIs (Fig. 3B). These results indicate that AIV H9N2 infection can clearly influence circMerTK expression. We further utilized the PK-15 cell line for the challenge with IAV PR8, and pseudorabies virus (PRV, dsDNA genome) infection as an example of a DNA virus at various time points. Interestingly, not only IAV PR8 but also PRV promoted circMerTK expression, as validated by both RT-PCR and RT-qPCR (Fig. 3C to F).

The A549 human cell line was then used to evaluate the circMerTK expression pattern after being challenged with Sendai virus (SeV, negative-stranded ssRNA genome) and Muscovy duck reovirus (MDRV, dsRNA genome) viruses. Similarly, the time-course study showed that circMerTK levels were elevated during SeV and MDRV infection (Fig. 3G and H). Taken together, these data suggest that circMerTK is a highly conserved circRNA induced in a wide variety of human and animal cell lines by diverse RNA and DNA viruses, suggesting that its expression is universal under a variety of viral infection conditions.

### CircMerTK expression is induced by IFN-β during IAV infection.

**FIG 4:**
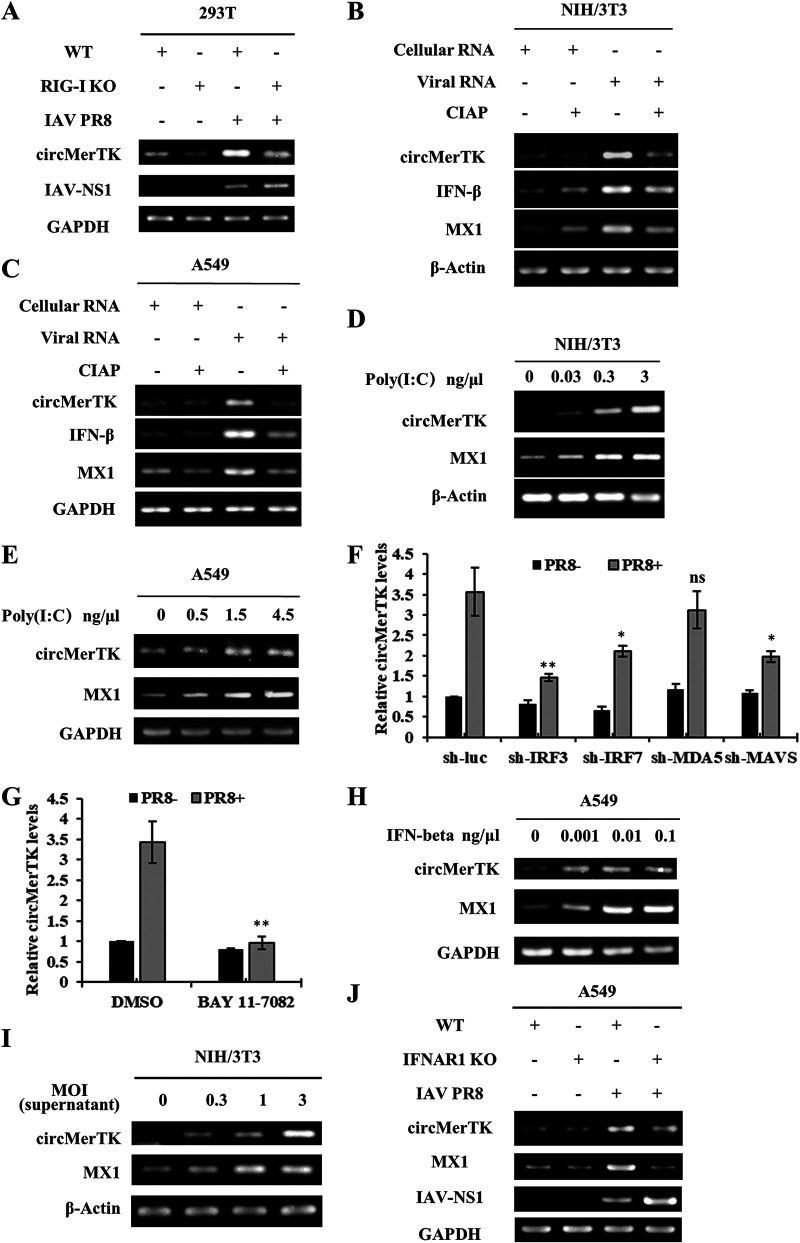
CircMerTK expression is induced by interferon β (IFN-β) during IAV infection. (A) IAV PR8 infected RIG-I knockout (KO) 293T cell line for 14 h, and the expression of circMerTK was examined by RT-PCR. (B) Total RNA from NIH/3T3 and (C) A549 cells infected with IAV PR8 (Viral RNA) or without infection (Cellular RNA), treated or not treated with calf intestinal alkaline phosphatase (CIAP), were transfected into native A549 and NIH/3T3 cells. Next, RNAs were extracted, and circMerTK expression was examined by RT-PCR. (D) NIH/3T3 and (E) A549 cell lines were transfected with poly(I:C) at indicated concentrations for 4 h. Then, the levels of circMerTK along with MX1 were examined by RT-PCR. (F) Interferon regulatory factor (IRF) 3, IRF7, antimelanoma differentiation-associated gene 5 (MDA5), and mitochondrial antiviral-signaling protein (MAVS) A549 knockdown cell lines were generated and challenged or not challenged with IAV PR8 infection for 14 h. circMerTK expression level was relatively quantified by RT-qPCR. (G) A549 cells were either treated with BAY 11–7082 or the vehicle (dimethyl sulfoxide, DMSO) and then infected with IAV PR8 or mock-infected for 14 h. RT-qPCR was used to assess the relative expression of circMerTK. (H) Expression of circMerTK was examined by RT-PCR in A549 cells treated with IFN-β at indicated concentrations for 2 h. (I) Native NIH/3T3 cell line was stimulated for 1 h with supernatants from NIH/3T3 cells infected or not infected with IAV PR8 at different MOIs for 14 h, and circMerTK expression was examined by RT-PCR. (J) Expression of circMerTK was examined by RT-PCR in IFNAR1 knockout A549 cell line infected with or without IAV PR8 for 14 h. Shown are representative data from three independent experiments. Data represent the mean values ± SD (*n* = 3; *, *P* < 0.05; **, *P* < 0.01; ns, not significant). WT, wild type.

To understand the induction mechanism of circMerTK by viral infection, we devised a series of experiments to pinpoint the signaling pathway that may regulate circMerTK expression upon IAV infection. Using CRISPR-Cas9, we generated a 293T RIG-I knockout (KO) cell line. The RIG-I knockout was confirmed by DNA sequencing, and deficiency of RIG-I expression was substantiated by Western blotting (Fig. S2A). Then, both RIG-I knockout and wild-type (WT) 293T cells were infected with IAV PR8. Unlike the WT control cells, RIG-I knockout cells were unable to upregulate circMerTK expression in response to IAV infection (Fig. 4A), suggesting that RIG-I-dependent innate immune signaling is required for efficient induction of circMerTK by the IAV infection.

To confirm this observation, A549 cells were transfected with viral RNA from IAV-infected cells or mock-infected cells and then treated with or without calf intestinal alkaline phosphatase (CIAP). Indeed, the viral RNA induced the expression of circMerTK, while CIAP treatment of viral RNA repressed circMerTK expression, as well as the production of IFN-β and MX1, in both NIH/3T3 and A549 cell lines (Fig. 4B and C). Next, multiple human and mouse cell lines were transfected with poly(I:C) at varying concentrations. Intriguingly, poly(I:C) clearly enhanced the expression of circMerTK in mouse (NIH/3T3, MLE-12) and human (A549, 293T) cell lines in a dose-dependent manner (Fig. 4D and E, Fig. S2B and C). These results imply that the induction of circMerTK expression during IAV infection is likely regulated by RIG-I-dependent IFN signaling.

To determine whether other pattern recognition receptors (PRRs) and signaling molecules are involved in the regulation of circMerTK expression, we employed several cell lines stably expressing short hairpin RNAs (shRNAs) specifically targeting PRRs or signaling molecules, as previously described (24–26). We observed that knockdown of interferon regulatory factor (IRF) 3, IRF7, and mitochondrial antiviral-signaling protein (MAVS) significantly reduced circMerTK levels following IAV infection, but knockdown of antimelanoma differentiation-associated gene 5 (MDA5) had no significant impact on circMerTK expression (Fig. 4F). In addition, BAY 11-7082, an inhibitor of the NF-κB signaling cascade, was used to study the relationship between NF-κB and circMerTK expression. Notably, BAY 11-7082 treatment also led to a significant reduction in circMerTK levels induced by IAV infection (Fig. 4G, Fig. S2D).

These findings imply that type I IFN signaling may regulate the production of circMerTK during IAV infection. To test this possibility, we examined directly whether IFN-β could induce circMerTK expression. Indeed, treatment of A549 cells with IFN-β caused a robust expression of circMerTK (Fig. 4H). Additionally, supernatants collected from NIH/3T3 cells infected with IAV at various MOIs for 14 h were used to stimulate naive NIH/3T3 cells for 1 h. As expected, NIH/3T3 cells stimulated with the supernatants had higher circMerTK levels than those in control cells (Fig. 4I). These findings were corroborated by experiments using IFNAR1 knockout A549 cells previously developed by our group (27, 28), in which type I IFN signaling was impaired. We found that circMerTK expression decreased compared to that in WT cells after IAV infection (Fig. 4J). Altogether, our findings indicate that IAV-induced circMerTK expression is regulated by innate immune signaling involving type I IFNs.

### Altering circMerTK expression has a substantial impact on IAV replication.

**FIG 5:**
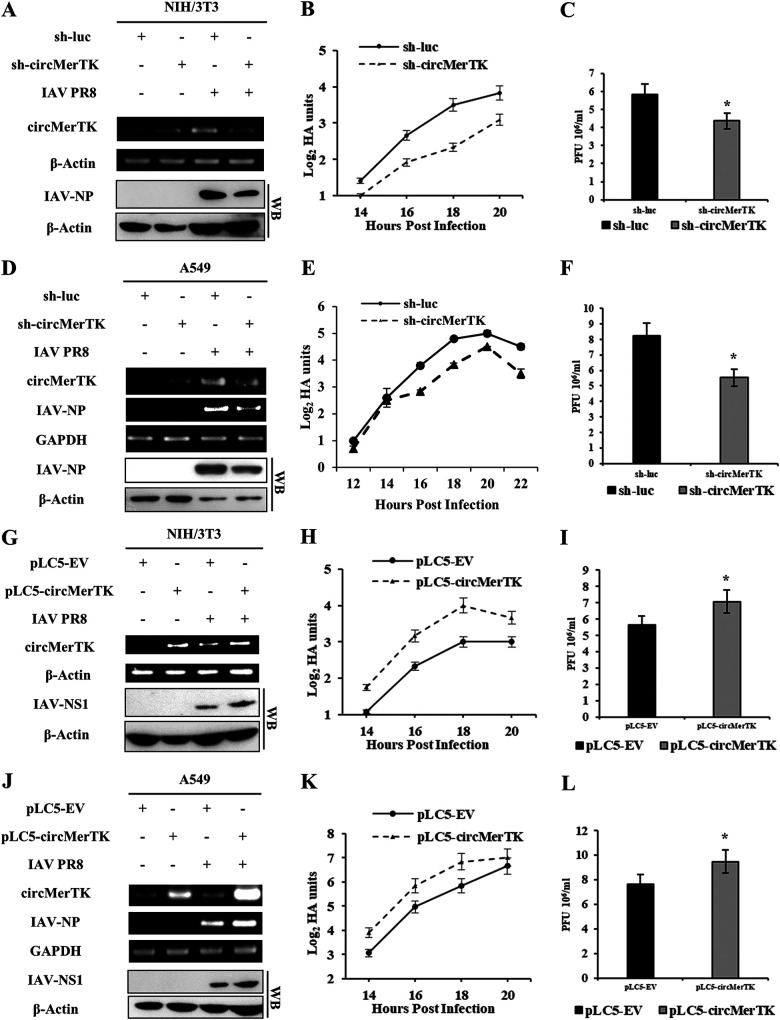
Altering circMerTK expression has a substantial impact on IAV replication. (A) NIH/3T3 cell lines stably expressing specific short hairpin RNAs (shRNAs) targeting circMerTK (sh-circMerTK) or luciferase (sh-luc, control) were infected with or without IAV PR8 and the knockdown efficiency was checked by RT-PCR. Western blotting was utilized to assess IAV-NP levels. (B) IAV titers in the supernatants of PR8-infected sh-circMerTK and sh-luc NIH/3T3 cells were measured at indicated time points by standard hemagglutination assay (HA) (MOI = 0.5). (C) Supernatants from PR8-infected sh-circMerTK or sh-luc NIH/3T3 cells were collected at 16 hpi (MOI = 0.5). IAV titers in supernatants were examined with plaque assay. (D) A549 cell lines stably expressing specific shRNAs targeting circMerTK or luciferase were infected with or without IAV PR8 and the knockdown efficiency was confirmed by RT-PCR. Western blotting was utilized to assess IAV-NP levels. (E) IAV titers in the supernatants of PR8-infected sh-circMerTK and sh-luc A549 cells were measured at indicated time points by HA assay (MOI = 0.5). (F) Supernatants from PR8-infected sh-circMerTK or sh-luc A549 cells were collected at 16 hpi (MOI = 0.5). IAV titers in the supernatants were examined with plaque assay. (G) RT-PCR was utilized to assess the overexpression efficiency of circMerTK in NIH/3T3 cells stably expressing pLC5-EV or pLC5-circMerTK following IAV PR8 infection. Western blotting was used to assess IAV-NS1 protein levels. (H) IAV titers in the supernatants of PR8-infected pLC5-circMerTK-overexpressing and pLC5-EV NIH/3T3 cells were measured at indicated time points by HA assay (MOI = 0.5). (I) Supernatants from PR8-infected pLC5-circMerTK-overexpressing and pLC5-EV NIH/3T3 cells were collected at 16 hpi (MOI = 0.5). IAV titers in the supernatants were examined with plaque assay. (J) RT-PCR was utilized to assess the overexpression efficiency of circMerTK in A549 cells stably expressing pLC5-EV or pLC5-circMerTK following IAV PR8 infection. Western blotting was utilized to assess IAV-NS1 protein levels. (K) IAV titers in the supernatants of PR8-infected pLC5-circMerTK-overexpressing and pLC5-EV A549 cells were measured at indicated time points by HA assay (MOI = 0.5). (L) Supernatants from PR8-infected pLC5-circMerTK-overexpressing or pLC5-EV A549 cells were collected at 16 hpi (MOI = 0.5). IAV titers in the supernatants were examined with plaque assay. Representative data from three independent experiments are shown. Data represent the mean values ± SD (*n* = 3; *, *P* < 0.05).

Since MerTK is known as a regulator of innate immunity against viral infection (16, 17), we sought to determine whether circMerTK derived from the pre-mRNA of MerTK possessed an immune function in response to IAV infection. For this, we used shRNAs to precisely silence circMerTK without altering the transcription of linear MerTK mRNA (Fig. S3A). We established a NIH/3T3 cell line stably expressing an shRNA targeting circMerTK. Transduction efficiency was validated by observing green fluorescent protein (GFP) expression using fluorescence microscopy, and the disruption of circMerTK expression and the relative quantification of MerTK mRNA expression were analyzed by RT-PCR and RT-qPCR in cells with or without IAV challenge (Fig. 5A, Fig. S3B to D). Hemagglutination assay (HA) titer curve analysis revealed that silencing circMerTK resulted in a substantially lower viral load than that in the control (Fig. 5B). A plaque assay analysis confirmed this finding, as sh-circMerTK cells displayed lower influenza titers than control cells (Fig. 5C).

Next, we investigated the effect of circMerTK on IAV infection in human lung epithelial cells. CircMerTK knockdown A549 cell lines were generated using shRNA targeting circMerTK (Fig. 5D, Fig. S3E and F). Similarly, analysis of viral titers by HA showed that knockdown of circMerTK in A549 cells resulted in decreased viral titers compared to the control (Fig. 5E). A plaque assay analysis further validated these observations (Fig. 5F). Together, these experiments demonstrated that silencing circMerTK inhibited IAV replication. Furthermore, we examined whether the antiviral activity of circMerTK is restricted to the influenza virus or has broad-spectrum antiviral effects. SeV virus was used to challenge circMerTK-silenced A549 cells along with the control cells. The RT-qPCR results revealed that circMerTK-silenced A549 cells had lower mRNA levels of SeV-nucleocapsid protein (SeV-NP) than the control cells (Fig. S3G). Consistently, HA analysis showed that the viral titers in sh-circMerTK cells infected with SeV were lower than those in control (Fig. S3H). Since our data suggested that circMerTK might have a comparable function to that of MerTK in enhancing viral replication, as described previously (16), and our results showed that silencing circMerTK had no effect on MerTK expression, we asked whether disruption of MerTK mRNA would affect circMerTK expression and influence its function. To address this question, we designed an shRNA to target the MerTK mRNA (Fig. S3I). The expression level of circMerTK was examined in control and sh-MerTK knockdown cell lines after IAV challenge using RT-PCR and RT-qPCR. Our data demonstrated that silencing MerTK expression did not affect circMerTK levels in cells challenged or not challenged with influenza virus (Fig. S3I and J).

On the other hand, we used a pLC5-ciR overexpression system to determine whether increasing circMerTK expression had any effects on IAV replication. To this end, NIH/3T3 cell lines overexpressing circMerTK were generated, and RT-PCR and RT-qPCR were utilized to confirm the expression levels of circMerTK and MerTK mRNA following challenge with or without IAV (Fig. 5G, Fig. S4A and B). As shown in Fig. 5H, circMerTK-overexpressing cells had greater viral titers than those in the control cells, as indicated by the HA curve. Consistent with this, plaque assay analysis demonstrated a substantial increase in the number of influenza plaques compared to the control (Fig. 5I). Furthermore, circMerTK overexpression A549 cell lines were generated (Fig. 5J, Fig. S4C and D), which also displayed greater IAV loads by HA than those in control cells following viral infection (Fig. 5K). Similarly, a plaque assay established that influenza titers were increased in the circMerTK overexpression cells compared to those in the control (Fig. 5L). Moreover, RT-qPCR revealed elevated expression of IAV nucleoprotein (IAV-NP) in the circMerTK-overexpressing A549 cells compared to those in the control (Fig. S4E). In addition, circMerTK overexpression in 293T cells also caused enhanced IAV replication, as evidenced by stronger influenza NP and nonstructural protein 1 (NS1) signals in these cells following the infection (Fig. S4F). Additionally, circMerTK-overexpressing A549 cells were subjected to a SeV viral challenge. Notably, the level of SeV-NP mRNA was significantly increased in circMerTK-overexpressing A549 cells compared with that in the control cells (Fig. S4G). Moreover, HA analysis of the supernatants of SeV-infected circMerTK-overexpressing cells showed higher viral loads than those in the control cells (Fig. S4H).

The results reveal that circMerTK is a virus-induced circular RNA that promotes influenza virus replication in both human and mouse cells. Similarly, it is also involved in enhancing SeV virus infection.

### Silencing circMerTK enhances the innate antiviral response, resulting in impaired expression of the IAV NP gene.

**FIG 6:**
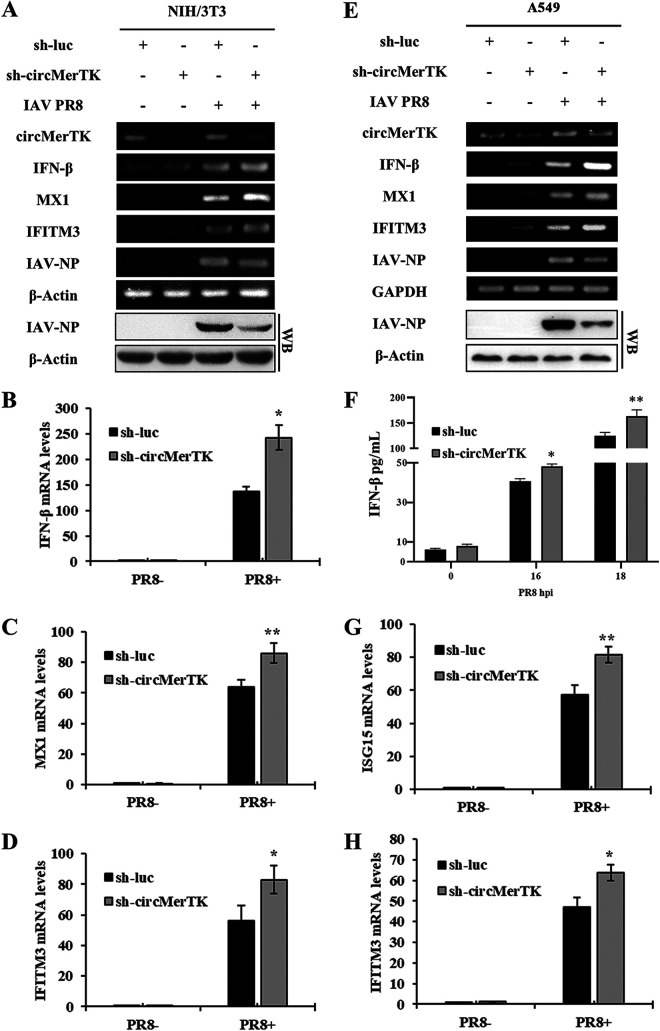
Silencing circMerTK enhances the innate antiviral response, resulting in impaired expression of the IAV NP gene. (A) NIH/3T3 cell lines stably expressing specific shRNAs targeting circMerTK or luciferase were infected with or without IAV PR8 for 14 h. IFN-β, MX1, and IFITM3 mRNA expression levels were determined by RT-PCR. Western blotting was utilized to assess IAV-NP levels. (B) mRNA levels of IFN-β, (C) MX1, and (D) IFITM3 were evaluated in circMerTK knockdown NIH/3T3 cell lines by RT-qPCR as described in panel A. (E) A549 cell lines stably expressing specific shRNAs targeting circMerTK or luciferase were infected with or without IAV PR8 for 14 h. IFN-β, MX1, and IFITM3 mRNA expression levels were determined by RT-PCR. Western blotting was utilized to assess IAV-NP levels. (F) circMerTK knockdown A549 cells, along with the control, were infected with IAV PR8 for 16 and 18 h. Enzyme-linked immunosorbent assay (ELISA) was used to examine IFN-β protein levels in the supernatants at the indicated time points. (G) mRNA levels of ISG15 and (H) IFITM3 were evaluated in circMerTK knockdown A549 cell lines by RT-qPCR as described in panel E. Representative data from three independent experiments are shown. Data represent the mean values ± SD (*n* = 3; *, *P* < 0.05; **, *P* < 0.01).

Our data shown above suggested that circMerTK expression is regulated by innate immune signaling during IAV infection and that this induction of circMerTK by IAV is favorable to viral replication. Therefore, we asked whether circMerTK played a role in the innate immune response to influenza infection. We challenged circMerTK-silenced and control cells with IAV PR8 and measured the expression of IFN-β and several key ISGs. The RT-PCR results showed that silencing circMerTK in NIH/3T3 cells caused increased expression of IFN-β, MX1, and IFITM3 mRNAs after IAV infection compared to the control (Fig. 6A). Consistently, Western blotting revealed lower levels of viral NP protein in circMerTK knockdown cells compared to those in the control cells (Fig. 6A). RT-qPCR further confirmed that NIH/3T3 cells expressing sh-circMerTK had enhanced expression of IFN-β, MX1, and IFITM3 mRNA (Fig. 6B to D).

Moreover, human A549 cell lines stably expressing sh-circMerTK also showed elevated levels of IFN-β, MX1, and IFITM3 mRNA after IAV PR8 infection compared to those in the control cells (Fig. 6E). This was consistent with the decreased viral NP level shown by Western blotting (Fig. 6E). Additionally, data from enzyme-linked immunosorbent assay (ELISA) of cell culture supernatants of the sh-circMerTK A549 cell line showed higher levels of IFN-β after IAV infection compared to the control (Fig. 6F). Similarly, RT-qPCR revealed that knockdown of circMerTK in A549 cells resulted in a significant increase in IFN-β, ISG15, and IFITM3 mRNAs (Fig. 6G and H, Fig. S5A). These observations indicate that circMerTK deficiency enhances the type I IFN antiviral response in both human and mouse cells and thereby impairs viral replication.

### Overexpression of circMerTK suppresses antiviral immunity, enhancing IAV NP gene expression.

**FIG 7:**
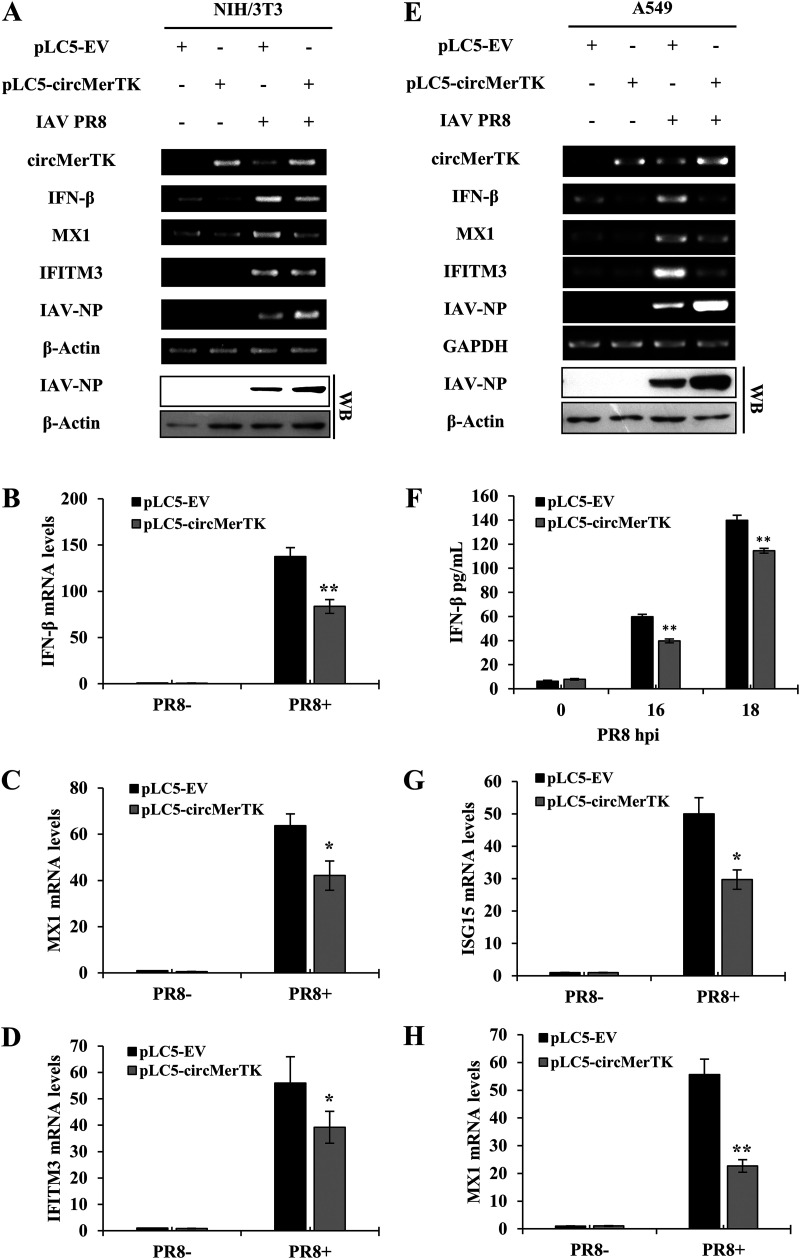
Overexpression of circMerTK suppresses antiviral immunity, enhancing IAV NP gene expression. (A) IFN-β, MX1, and IFITM3 mRNA expression levels were detected in pLC5-EV and pLC5-circMerTK NIH/3T3 cell lines with or without IAV PR8 infection at 14 hpi by RT-PCR. Western blotting was used to assess IAV-NP levels. (B) Expression levels of IFN-β, (C) MX1, and (D) IFITM3 were evaluated in pLC5-EV and pLC5-circMerTK NIH/3T3 cell lines with or without IAV PR8 infection by RT-qPCR. (E) mRNA expression levels of IFN-β, MX1, and IFITM3 in the pLC5-EV and pLC5-circMerTK A549 cell lines with or without IAV PR8 infection at 14 hpi were determined by RT-PCR. Western blotting was used to assess IAV-NP levels. (F) circMerTK-overexpressing A549 cells and control pLC5-EV cells were infected with IAV PR8 for 16 and 18 h. ELISA was used to quantify IFN-β protein levels in the supernatants at the indicated time points. (G) Expression levels of ISG15 and (H) MX1 were evaluated in pLC5-EV and pLC5-circMerTK A549 cell lines with or without IAV PR8 infection by RT-qPCR. Representative data from three independent experiments are shown. Data represent the mean values ± SD (*n* = 3; *, *P* < 0.05; **, *P* < 0.01).

We next employed an overexpression system to further investigate the involvement of circMerTK in the innate antiviral response against IAV infection. For this, we generated cell lines overexpressing circMerTK and evaluated the levels of IFN-β and several critical ISGs. Indeed, overexpression of circMerTK in NIH/3T3 led to a decline in IFN-β mRNA production and decreased expression of critical ISGs such as MX1 and IFITM3, which was associated with the increased expression of viral NP mRNA and protein (Fig. 7A). RT-qPCR results validated this finding, showing significantly decreased levels of IFN-β, MX1, and IFITM3 mRNA (Fig. 7B to D).

Likewise, RT-PCR demonstrated that A549 cell lines overexpressing circMerTK had diminished levels of IFN-β, MX1, and IFITM3 mRNA. This was coupled with enhanced expression of viral NP at both the mRNA and protein levels (Fig. 7E). Furthermore, quantification of IFN-β by ELISA in cell culture supernatants demonstrated significantly suppressed production of IFN-β in the circMerTK overexpression cell line compared to the control (Fig. 7F). RT-qPCR substantiated this observation, demonstrating that overexpression of circMerTK reduced the levels of IFN-β, ISG15, and MX1 mRNA following viral infection compared to the empty vector control (Fig. 7G and H, Fig. S5B).

Next, we sought to investigate the mechanism of circMerTK’s innate immune activity. Utilizing CircAtlas, an online repository for circRNAs interaction predictions (29), we found that several microRNAs are predicted to interact with circMerTK. Particularly, CircAtlas predicted that circMerTK might sponge miR-125a-3p, a critical microRNA that has been shown to be involved in the p38/MAPK pathway affecting cellular apoptosis (30). We devised exploratory experiments and observed that a miR-125a-3p mimic reduced the circMerTK level, while A549 cells treated with miR-125a-3p inhibitor had a higher circMerTK expression level (Fig. S6A and B). However, the interaction between circMerTK and miR-125a-3p and the role of this interaction in innate antiviral immunity remains to be determined. In summary, our data establish circMerTK as a crucial circular RNA that regulates IFN-β production and the downstream induction of essential antiviral ISGs.

## DISCUSSION

CircRNAs are an exceptional class of RNAs characterized by a unique circular structure due to specific back-splicing mechanisms which cause the 5′ and 3′ RNA ends to join (6). Despite numerous studies attempting to investigate the functions of circRNAs, investigation of the roles of circRNAs in innate immunity against viral infection is still an ongoing task. A better understanding of the underlying mechanism of circRNA-virus interaction will provide innovative approaches for studying and developing new targets for drug development and diagnostic biomarkers. CircRNAs are emerging as shining new stars for the diagnosis and cure of various illnesses and cancers (31–33). This subclass of noncoding RNAs functions by a variety of mechanisms, including miRNA sponging, protein binding, gene transcription or splicing regulation, epigenetics, and encoding proteins or peptides (34).

Previous studies on anti-influenza innate immunity have attempted to elucidate the roles of circRNAs in the innate antiviral response to influenza virus infection. Some circRNAs have been shown to function as antiviral circRNA molecules, whereas others have been shown to enhance influenza virus replication and infection. For instance, it has been reported that circRNA_0050463 and circ-GATAD2A facilitate and accelerate influenza virus replication through distinct mechanisms (11, 12). In contrast, it has been observed that circRNA AIVR restricts influenza virus replication by facilitating IFN-β production (13). However, the information available on the functional involvement of circRNAs in IAV pathogenesis is still limited.

In this study, we intended to uncover key circRNA(s) involved in the IAV-host interaction, thereby elucidating some of the dynamics associated with influenza pathogenesis, which might lead to the identification of potential therapeutic targets or biomarkers for controlling influenza. Here, we identified circMerTK, a novel circRNA with a 275-base-long exonic circRNA produced by back-splicing of MerTK pre-mRNA exons 3 and 4. It is listed under the following circBase IDs: hsa_circ_0056121 for the human homolog, and mmu_circ_0009326 for the mouse homolog. We decided to focus principally on studies of circMerTK for several reasons: (i) its expression was highly induced by viral infection *in vivo*, (ii) it is derived from the pre-mRNA of a crucial gene that has been previously implicated in innate immunity (16, 17, 23), and (iii) it is highly conserved across mammals, with homologs in Homo sapiens (humans), Macaca mulatta (rhesus macaques), Mus musculus (mice), and Canis lupus (dogs); in particular, it shares a high identity of 86% between the human and mouse homologs.

Our results reveal that circMerTK is induced both *in vivo* and *in vitro* by IAV infection in a wide range of human and animal cell lines. Interestingly, previous studies have suggested that the conservation of circular RNA generation would indicate the evolutionary preservation of circular RNA formation and a possibly vital role (4, 35). Importantly, several DNA and RNA viruses, including IAV PR8 and WSN, AIV H9N2, SeV, MDRV, and PRV, upregulated the expression of circMerTK *in vitro*. This observation suggests that circMerTK may generally play a critical role in regulating innate immune responses. This agrees with several investigations which showed that some circRNAs can be induced by viral infections and are involved in innate immunity (31). However, the functional involvement of circMerTK in the pathogenesis of other viruses and the precise underlying mechanisms remain to be determined. On the other hand, it would be interesting to test whether circMerTK is upregulated in clinical samples from virus-infected patients. This requires further investigation.

Furthermore, we observed that RIG-I knockout A549 cells failed to upregulate circMerTK expression following IAV infection. In addition, viral RNA promoted the expression of circMerTK, while CIAP-treated viral RNA failed to stimulate circMerTK expression, indicating that RIG-I pattern recognition receptor is involved in the induction of circMerTK. Also, poly(I:C) induced circMerTK expression in various animal and mouse cell lines in a dose-dependent manner. Consistently, IFN-β treatment of A549 cells boosted levels of circMerTK expression. This was subsequently validated using IFNAR1 knockout cells, which demonstrated diminished expression of circMerTK following the viral infection. A previous study showed that treating human monocyte-derived macrophages with poly(I:C), IFN-α, and IFN-γ suppressed MerTK expression (36); other investigations have established that circRNA synthesis competes with canonical pre-mRNA splicing, which may explain this phenomenon (37, 38).

Functional analysis indicated that circMerTK enhances IAV and SeV replication, as overexpression of circMerTK increased the viral replication titer, which was associated with a decrease in IFN-β production and suppression of type I IFN signaling. In contrast, silencing circMerTK negatively affected viral replication, manifested by lower virus replication titers and increased IFN-β and ISG production. CircRNAs are reported to function via several mechanisms, interfering with multiple signaling pathways by affecting the target molecules. Importantly, circRNAs can effectively sponge microRNAs and act as competing endogenous RNAs to interfere with microRNA function. Our preliminary investigation suggested that miR-125a-3p could interact with circMerTK. This agrees with a recent study showing that circMerTK could sponge miR-125a-3p (39). Nevertheless, the interplay between circMerTK and miR-125a-3p and its functional involvement in innate immunity requires additional study.

circMerTK is an exonic circRNA derived from MerTK pre-mRNA. *In vivo* and *in vitro* studies have established that linear MerTK can modulate innate immunity against various viral infections. For instance, MerTK signaling following VSV infection was shown to upregulate the expression of suppressor of cytokine signaling 1 (SOCS1) and SOCS3 *in vivo*, which was associated with a decline in type I IFN production (17). MerTK was also reported to facilitate the entry of CSFV by interacting with the E2 protein of CSFV, in addition to downregulating the expression levels of IFN-β and contributing to BVDV infection (16). Although MerTK and circMerTK have comparable innate immune outcomes that dampen IFN-β production and favor virus replication, the relationship between circMerTK and the linear MerTK remains obscure.

In summary, circMerTK is a virus-induced circRNA involved in negatively regulating innate antiviral immunity against IAV and SeV, thereby enhancing the viral replication. In particular, circMerTK suppresses IFN signaling and the subsequent production of several critical ISGs. The precise mechanism by which circMerTK regulates antiviral responses needs to be further investigated.

## MATERIALS AND METHODS

### Ethics statement.

The animal experimental design and protocols were reviewed and approved by the Research Ethics Committee of the College of Animal Sciences (College of Bee Science), Fujian Agriculture and Forestry University. All experimental mouse procedures were carried out in accordance with the Regulations for the Administration of Affairs Concerning Experimental Animals approved by the State Council of the People’s Republic of China. The protocol implemented in this study was also in accordance with the guidelines contained in the International Guiding Principles for Biomedical Research Involving Animals (40).

### Viruses, viral infection, and virus titer assays.

As previously described by Maarouf et al. (24), the influenza A virus strains A/WSN/1933 (H1N1) (WSN), A/Puerto Rico/8/34 (H1N1) (PR8), A/chicken/Jiangsu/C4258/2012 (H9N2), and Sendai virus (SeV) were propagated in specific pathogen-free embryonated chicken eggs. In addition, pseudorabies virus (PRV) strain Min-A was propagated in Madin-Darby canine kidney (MDCK) cells, while MDRV was propagated in Vero cells as described previously (41). As described previously by Rai et al. (42), monolayers of different cell lines were infected with various viruses at the specified multiplicity of infection (MOI). For analysis of virus titers, cell supernatants were collected at the indicated time after viral infection. IAV titers in the supernatant, which had gone through serial dilutions, were determined by a standard plaque assay using MDCK cells or hemagglutination assay (HA) as previously described (43).

### RNA extraction, RT-PCR, and RT-qPCR.

RNA extraction was performed as previously described by Chen et al. (44). Briefly, the total RNA of cells or mice tissues was extracted by TRIzol reagent (Tiangen, China). Five μg total RNAs was reverse-transcribed into cDNA using Random Primer (TaKaRa Bio Inc., Japan) by GoScript Reverse Transcriptase (Promega, Madison, WI), followed by PCR using *Taq* DNA polymerase (TaKaRa) or quantitative PCR using KAPA SYBR FAST qPCR Kits (Roche, Indianapolis, IN). The oligonucleotides used in this study are listed in Table S1 in the supplemental material.

### RNA-Seq analysis.

Ten SPF C57BL/6 mice were divided into two groups (mock-infected and IAV PR8-infected). The infected group was inoculated with 1 × 10^4^ PFU/mL of IAV PR8 viral stock through intranasal instillation. Mice were euthanized, and lung samples were collected 48 h postinfection (hpi). CircRNA transcriptomes in mouse lungs were analyzed through RNA-Seq by Shanghai OE Biotech Co., Ltd. (Shanghai, China) using an Illumina NovaSeq 6000 instrument. The Gene Expression Omnibus (GEO, https://www.ncbi.nlm.nih.gov/geo/) accession number for the RNA-Seq data used in this paper is GSE210688. For CircRNA prediction and expression analysis, the company generated Sequence Alignment Map (SAM) files and aligned the sequencing reads of each sample with the reference genome using Burrows-Wheeler Aligner (BWA) software (45). Then, using CIRI software (46), and during the initial scanning of the SAM alignment, CIRI software identified junction reads with PCC (paired chiastic clipping) signals corresponding to circRNA candidates. Paired-end mapping (PEM) and GT-AG splicing signals at junctions were employed for preliminary filtering. After clustering junction reads and recording each circRNA candidate, CIRI analyzed the SAM alignment to seek additional junction reads one more time. In the meantime, CIRI conducted additional filtering to eliminate false-positive candidates caused by wrongly mapped reads of homologous genes or repetitive sequences. Schematic representation of circMerTK was performed using circPrimer 2.0 (47).

### Cell lines, cell culture, and cell stimulation.

Human lung epithelial cells (A549), human embryonic kidney cells (HEK293T/293T), mouse embryo fibroblast cells (NIH/3T3), murine lung epithelial-12 cells (MLE-12), mouse breast cancer cells (4T1), mouse lung adenoma epithelial-4 cells (LA-4), porcine kidney epithelial cells (PK-15), mouse mononuclear macrophage cells (RAW 264.7), chicken embryonic fibroblast cells (DF-1), and MDCK cells were cultured in Dulbecco’s modified Eagle’s medium (DMEM) (Gibco, Billings, MT) supplemented with 10% (vol/vol) fetal bovine serum (Gibco), 100 U/mL penicillin, and 100 U/mL streptomycin in 37°C incubator under humidified 5% CO_2_ as previously described (48). Recombinant human IFN-β was purchased from PeproTech (PeproTech Inc., Rocky Hill, NJ), and poly(I:C), BAY 11-7082, and dimethyl sulfoxide (DMSO) were purchased from Sigma-Aldrich (Sigma-Aldrich, St. Louis, MO). Cell stimulation was carried out as previously described (25). For viral RNA and cellular RNA stimulation, total RNA was extracted from IAV PR8 mocked or infected A549 or NIH/3T3 cells using TRIzol reagent (Tiangen, China) according to the manufacturer’s instructions. CIAP (TaKaRa Bio Inc., Japan) was used to dephosphorylate viral triphosphate RNA. A549 or NIH/3T3 cells were then transfected with these RNAs and tested as previously described (49). The miR-125a-3p mimic, mimic-NC, miR-125a-3p inhibitor, and inhibitor-NC were synthesized by RiboBio (Guangzhou, China) and transfected into A549 cells as previously described by Xiao et al. (50).

### Construction of plasmids and generation of stable cell lines.

The stable circMerTK knockdown or overexpression cell lines were generated by infecting cells with lentivirus harboring specific shRNA or circMerTK sequences in pSIH-H1-GFP or pLC5-ciR circRNA overexpression vector (Geneseed, China) vectors, respectively, as described previously (25, 43). The A549 and NIH/3T3 stable cell lines were utilized after a uniform GFP-positive population was obtained following 2 to 3 μg/mL puromycin (Sigma-Aldrich, St Louis, MO, P8833) treatment according to the manufacturer’s instructions. Sequences of shRNAs are listed in Table S1.

### Western blotting and antibodies.

Cell lysates were separated on SDS-PAGE, transferred onto nitrocellulose membrane, and probed with indicated antibodies for Western blotting as described previously (51). Anti-influenza A NS1 and anti-β-actin (Santa Cruz Biotechnology, Santa Cruz, CA) were used in this study. Anti-IAV NP polyclonal antibody was obtained by immunizing rabbits with GST-tagged NP protein as previously described (52).

### Generation of RIG-I and IFNAR1 knockout cell lines.

The CRISPR-Cas9 system was used as previously described by Liu et al. (27). In short, the Cas9 design target tool (https://zlab.bio/guide-design-resources) was used to design sgRNA sequences. After digesting the oligonucleotide pairs with BbsI (Thermo Fisher Scientific, Waltham, MA), the pair of annealed oligonucleotides was inserted into pSpCas9 (BB)-2A-GFP (PX458) plasmid (53). Using Lipofectamine 2000 (Invitrogen, Carlsbad, CA), the recombinant plasmids were transfected into the target cells. Two days after transfection, single cells were sorted using flow cytometry to assist in the development of single colonies. Table S1 lists the sequences utilized in the sgRNAs targeting the human RIG-I and IFNAR1 genes.

### ELISA.

The cell culture supernatants from the indicated human cell lines were collected, and IFN-β production was quantified using a Human IFN-β ELISA kit according to the manufacturer's instructions (Novus Biologicals, Littleton, CO).

### Statistical analysis.

Statistical analysis was performed using GraphPad Prism version 6.0 (GraphPad Software Inc.). *P* values were calculated predominantly using Student’s *t* test or, when mentioned, using a one-way analysis of variance (ANOVA) test. *P* ≤ 0.05 was considered significant. All data represent the mean values ± standard deviation.

### Data availability.

All data generated or used during the study appear in the submitted article. The GEO (https://www.ncbi.nlm.nih.gov/geo/) accession number for the RNA-Seq data used in this paper is GSE210688. Some data are available from the corresponding author by request. Further inquiries can be directed to the corresponding author.
