# Supplementary material for: Influenza A Virus-Induced circRNA circMerTK Negatively Regulates Innate Antiviral Responses
Source: Microbiol Spectr. 2023 Feb 27;11(2):e03637-22. doi: 10.1128/spectrum.03637-22 (PMC10100971; doi:10.1128/spectrum.03637-22)
Supplement: Supplemental file 1 — Supplemental material. Download spectrum.03637-22-s0001.pdf, PDF file, 0.8 MB [file spectrum.03637-22-s0001.pdf]

Supplementary figure 1

A

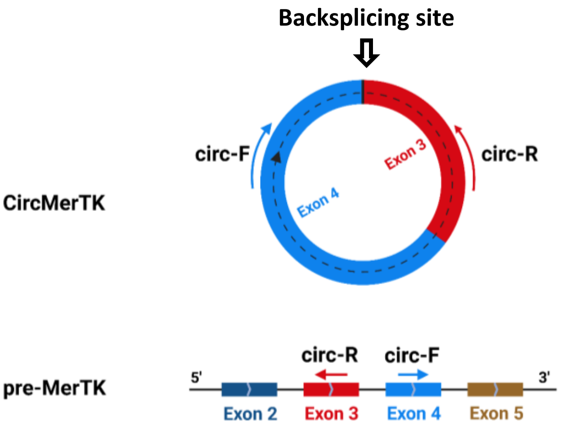

B

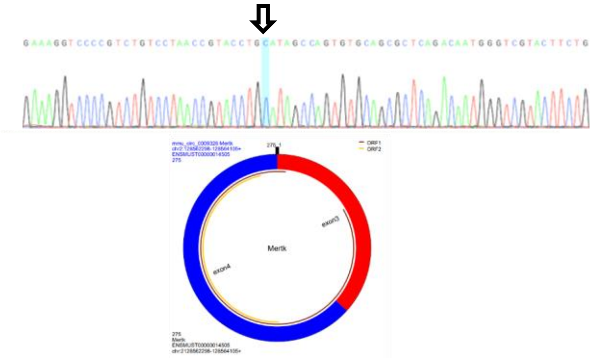

C

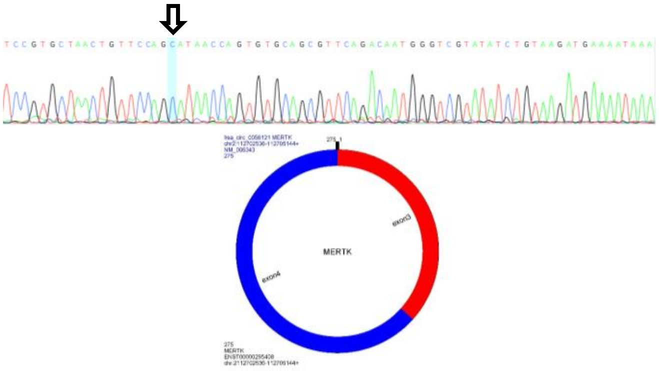

D

Score	Expect	Identities	Gaps	Strand
322 bits(356)	7e-93	236/275(86%)	0/275(0%)	Plus/Plus

```
hsa-circMerTK  1    CATAACCAGTGTGCAGCGTTCAGACAATGGGTCGTATATCTGTAAGATGAAAATAAACAA  60
                    |||| ||||||||||||| ||||||||||||||||| |||||||||||||  | |||||
mmu-circMerTK  1    CATAGCCAGTGTGCAGCGCTCAGACAATGGGTCGTACTTCTGTAAGATGAAGGTGAACAA  60

hsa-circMerTK  61   TGAAGAGATCGTGTCTGATCCCATCTACATCGAAGTACAAGGACTTCCTCACTTTACTAA  120
                    |  |||||| || ||||||||||| ||| | ||||| |||||||| ||| |||||| |||
mmu-circMerTK  61   TAGAGAGATTGTATCTGATCCCATATACGTGGAAGTTCAAGGACTCCCTTACTTTATTAA  120

hsa-circMerTK  121  GCAGCCTGAGAGCATGAATGTCACCAGAAACACAGCCTTCAACCTCACCTGTCAGGCTGT  180
                    ||||||||||||  ||||||||||||||||||||||||||||||||||||| ||||| ||
mmu-circMerTK  121  GCAGCCTGAGAGTGTGAATGTCACCAGAAACACAGCCTTCAACCTCACCTGCCAGGCCGT  180

hsa-circMerTK  181  GGGCCCGCCTGAGCCCGTCAACATTTTCTGGGTTCAAAACAGTAGCCGTGTTAACGAACA  240
                    |||||| |||||||||||||| || |||||||||||||| || ||||||||||| ||| |
mmu-circMerTK  181  GGGCCCTCCTGAGCCCGTCAATATCTTCTGGGTTCAAAATAGCAGCCGTGTTAATGAAAA  240

hsa-circMerTK  241  GCCTGAAAAATCCCCCTCCGTGCTAACTGTTCCAG  275
                     || ||||  ||||| || || ||||| || || |
mmu-circMerTK  241  ACCGGAAAGGTCCCCGTCTGTCCTAACCGTACCTG  275
```

FIG S1 (A) Schematic illustration of the circMerTK structure and the design strategy for the divergent primers (circ-F, circ-R) to target only the circular RNA and not the linear mRNA or pre-MerTK. (B) The schematic drawing represents the mouse homolog, and (C) the human homolog of circMerTK formed by backsplicing between exon 3 and exon 4 of MERTK pre-mRNA. The top part is the Sanger sequencing data; the arrow points at the backsplicing junction site. Schematic representation was generated by circPrimer2.0. (D) A multiple sequence alignment of human circMerTK (hsa-circMerTK) and the mouse circMerTK homolog (mmu-circMerTK), was made using basic local alignment search tool (BLAST, https://blast.ncbi.nlm.nih.gov/Blast.cgi).

Supplementary figure 2

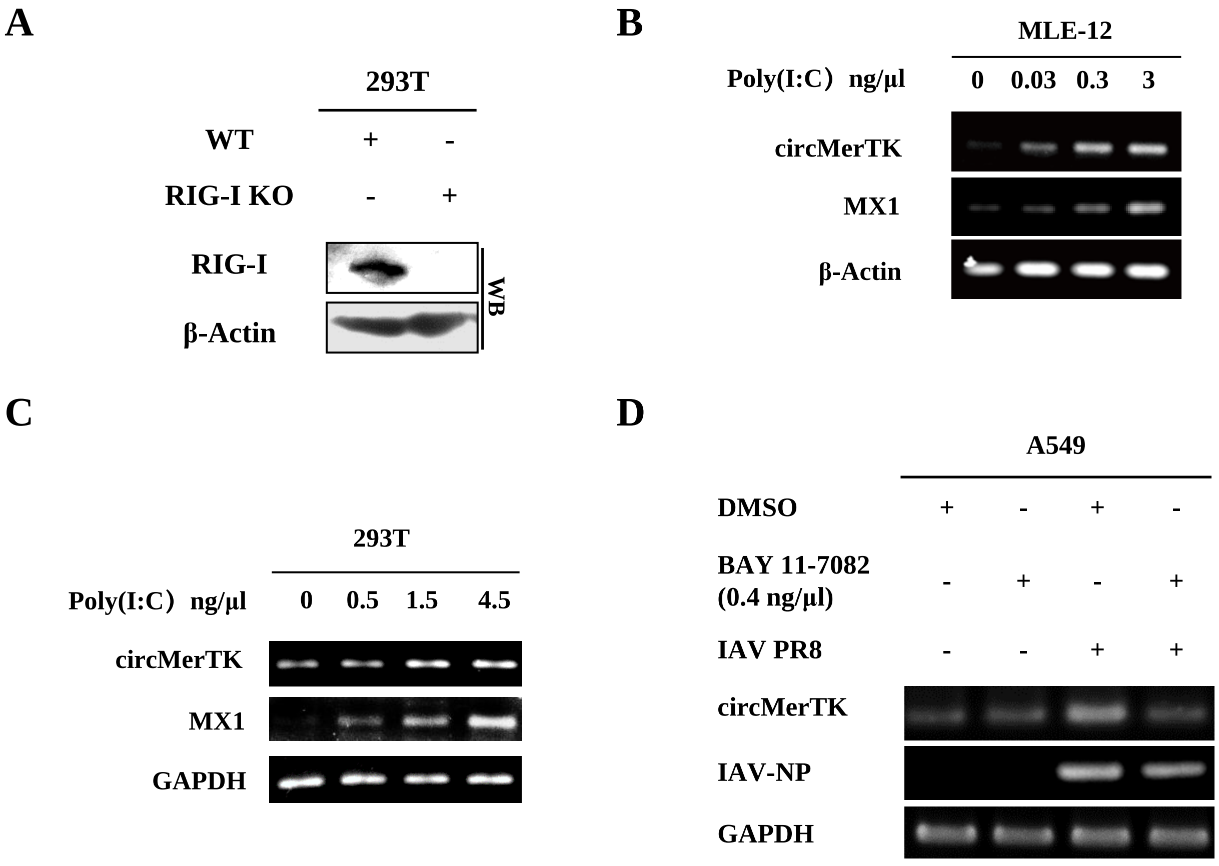

FIG S2 (A) Wild type (WT) 293T and RIG-I knockout (KO) 293T cell lines were examined for RIG-I protein expression by western blot. (B) MLE-12, and (C) 293T cell lines were transfected with poly(I:C) at indicated concentrations for 4 h. Then, the levels of circMerTK and MX1 were analyzed by RT-PCR. (D) A549 cells were either treated with BAY 11-7082 or DMSO and then infected with IAV PR8 or mock-infected for 14 hours. RT-PCR assessed the expression of circMerTK. Shown are representative data from three independent experiments. IAV-NP: IAV nucleoprotein; WB: western blot.

Supplementary figure 3

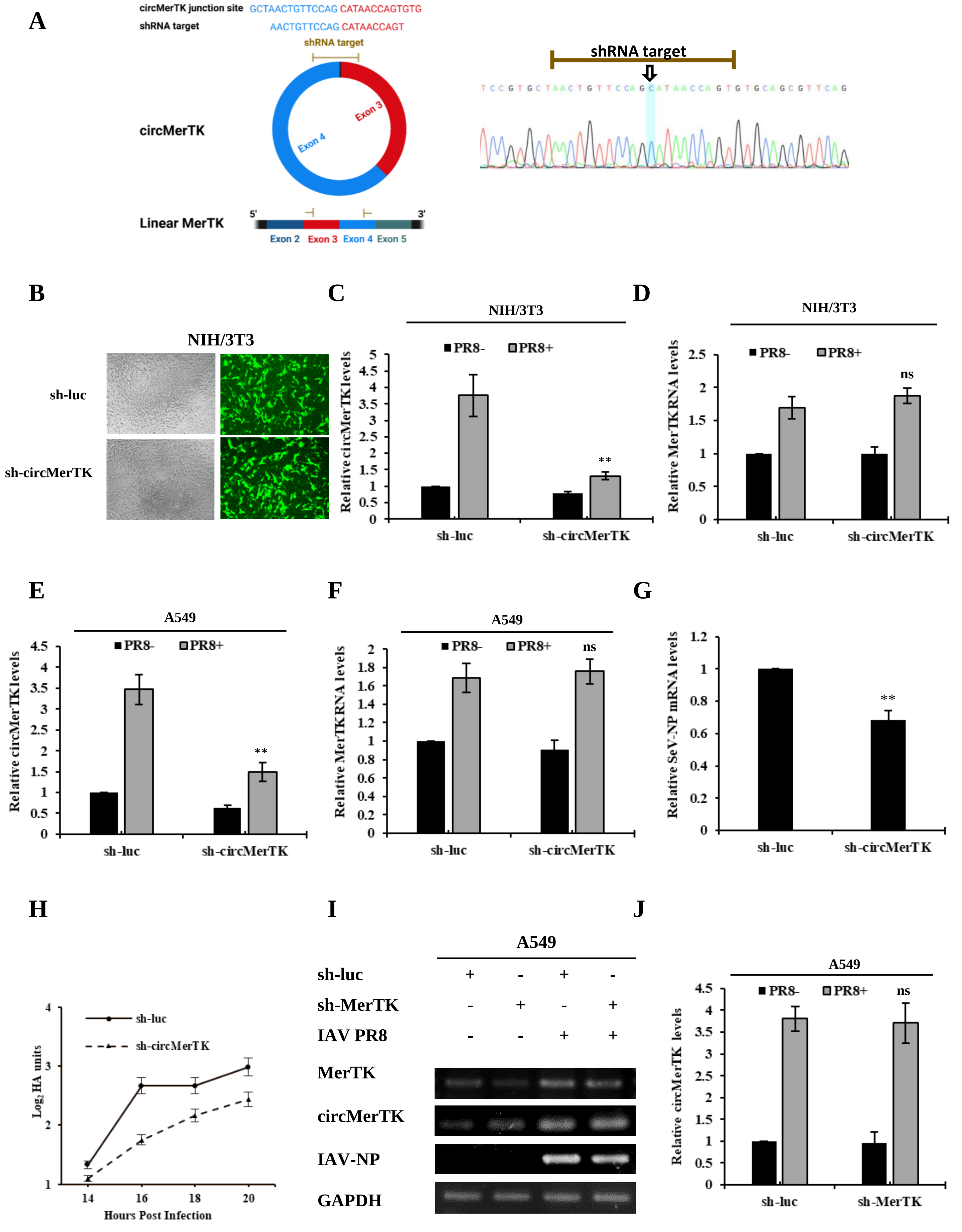

FIG S3 (A) Schematic illustration of the shRNA designed to target the human circMerTK without altering the expression of linear MerTK. (B) The transduction efficiency of NIH/3T3 cells stably expressing pSIH-H1-GFP vectors targeting circMerTK or luciferase control, was measured by visualizing the green fluorescent protein (GFP) fluorescence activity. (C-D) The expression levels of circMerTK and MerTK mRNA were assessed in sh-luc and sh-circMerTK NIH/3T3 cell lines by RT-qPCR. (E-F) The expression levels of circMerTK and MerTK mRNA were relatively quantified in sh-luc and sh-circMerTK A549 cell lines by RT-qPCR. (G) RT-qPCR was used to determine the mRNA levels of Sev-NP after SeV infection in sh-luc, and sh-circMerTK A549 cell lines for 16 hours (MOI = 0.5). (H) SeV titers in supernatants of SeV-infected sh-luc and sh-circMerTK A549 knockdown cells were measured at indicated time points by HA assay (MOI = 0.5). (I-J) The expression levels of circMerTK and MerTK mRNA were assessed in sh-luc and sh-MerTK A549 cell lines by RT-PCR and RT-qPCR. Results are shown as representative of three independent experiments with similar results. Data represent the mean values ± SD (n=3; ns= not significant, $^{**}p < 0.01$). IAV-NS1: IAV non-structural protein 1; IAV-NP: IAV nucleoprotein; SeV-NP: SeV nucleocapsid protein; WB: western blot.

Supplementary figure 4

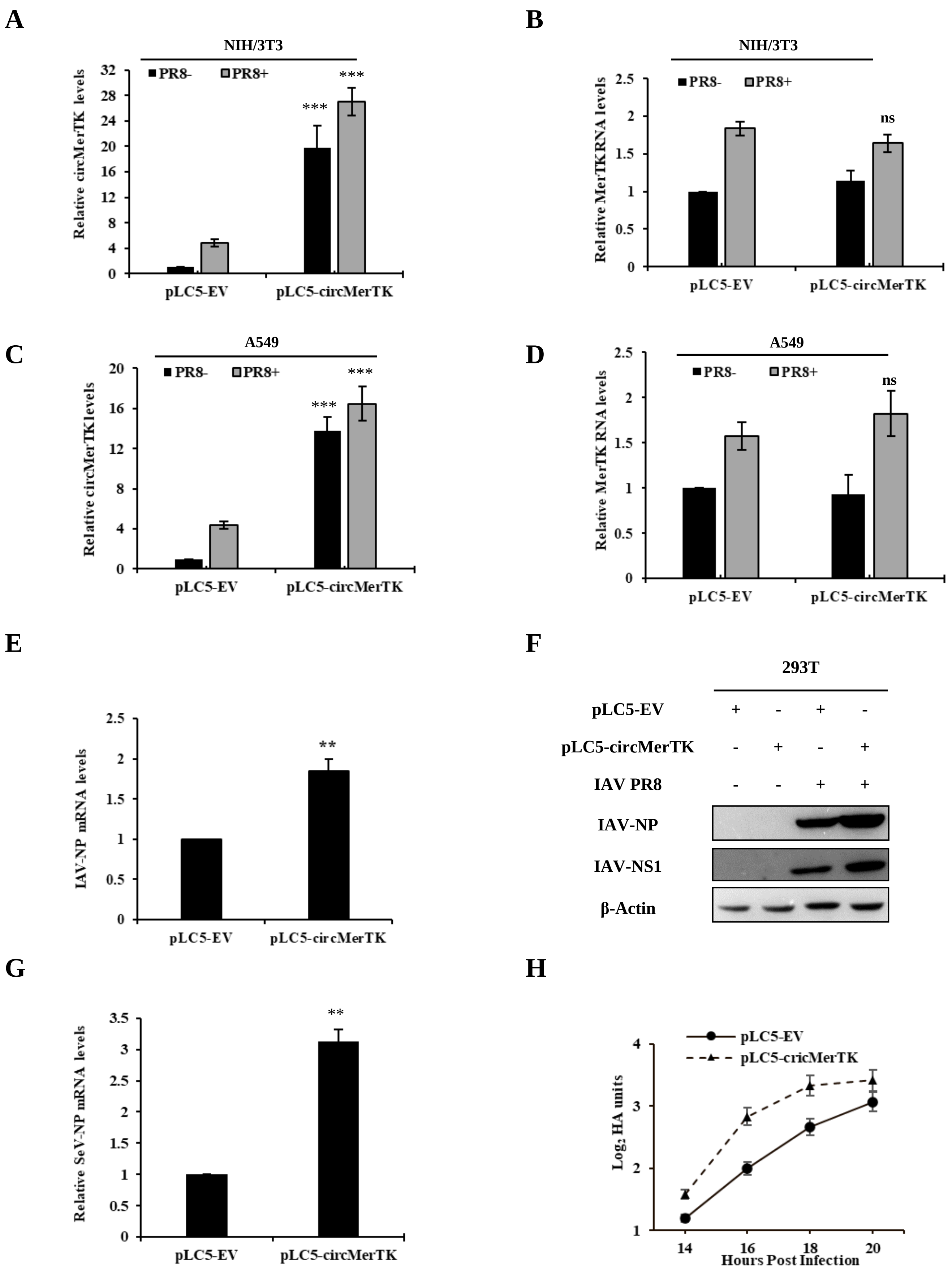

FIG S4 (A-B) The circMerTK and MerTK mRNA expression levels were assessed in pLC5-EV and pLC5-circMerTK NIH/3T3 cell lines by RT-qPCR. (C-D) The circMerTK and MerTK mRNA expression levels were evaluated in pLC5-EV and pLC5-circMerTK A549 cell lines by RT-qPCR. (E) RT-qPCR was used to determine the mRNA levels of IAV-NP after IAV PR8 infection in pLC5-EV, and pLC5-circMerTK stably expressed A549 cell lines for 16 hours (MOI = 0.5). (F) Western blotting was utilized to determine the protein levels of IAV-NP and IAV-NS1 after IAV PR8 infection of 293T stably expressing pLC5-EV or pLC5-circMerTK. (G) RT-qPCR was used to determine the mRNA levels of Sev-NP after SeV infection in pLC5-EV, and pLC5-circMerTK A549 cell lines for 16 hours (MOI = 0.5). (H) SeV titers in supernatants of SeV-infected pLC5-EV and pLC5-circMerTK A549 overexpression cells were measured at indicated time points by HA assay (MOI = 0.5). Results are shown as representative of three independent experiments with similar results. Data represent the mean values ± SD (n=3; ns= not significant, **$p < 0.01$,***$p < 0.001$). IAV-NS1: IAV non-structural protein 1; IAV-NP: IAV nucleoprotein; SeV-NP: SeV nucleocapsid protein; WB: western blot.

Supplementary figure 5

A **B**

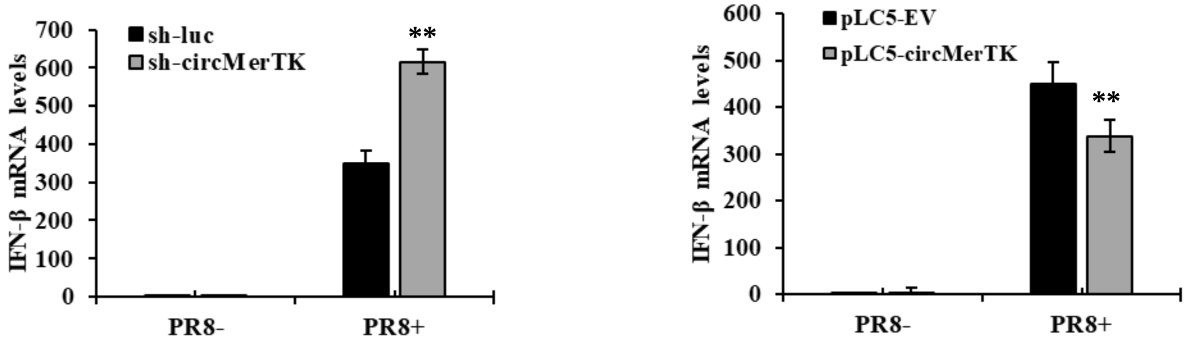

FIG S5 (A) A549 cell lines stably expressing specific shRNAs targeting circMerTK or luciferase were infected with or without IAV PR8 for 14 h. IFN-β mRNA expression levels were relatively quantified by RT-qPCR. (B) A549 cell lines stably expressing pLC5-EV or pLC5-circMerTK were challenged with or without IAV PR8 for 14 h. IFN-β mRNA expression levels were relatively quantified by RT-qPCR. Data represent mean ± SD (n=3; **p < 0.01).

Supplementary figure 6

A

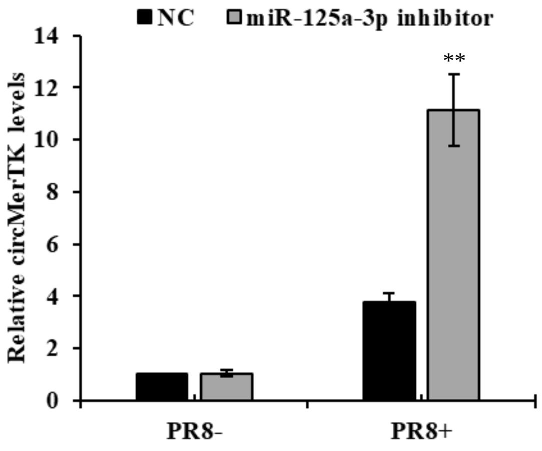

B

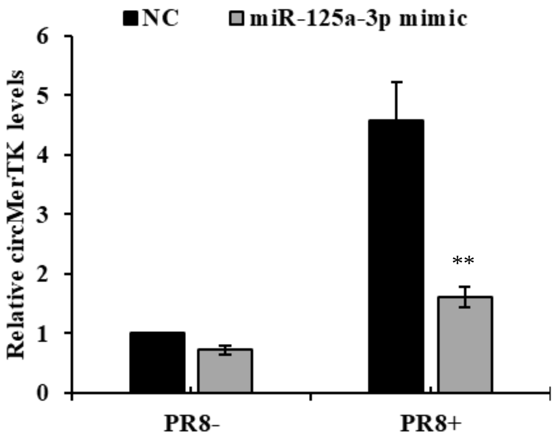

FIG S6 (A) A549 cell lines were transfected with either negative control (NC) or miR-125a-3p inhibitor for 24 h, followed by challenging both cell lines with IAV PR8 infection for 14h. The expression levels of circMerTK were determined by RT-qPCR in challenged and unchallenged cell lines. (B) A549 cell lines were transfected with either negative control (NC) or miR-125a-3p mimic for 24 h, followed by challenging both cell lines with IAV PR8 infection for 14h. The expression levels of circMerTK were determined by RT-qPCR in challenged and unchallenged cell lines. Data represent mean ± SD (n=3; **p < 0.01).

Supplementary table 1

mRNA Primers	
M-β-Actin-F	5’-AATGGGTCAGAAGGACTCCT-3’
M-β-Actin-R	5’-ACGGTTGGCCTTAGGGTTCAG-3’
M-GAPDH-F	5’-GCCTCGTCCCGTAGACAAAA-3’
M-GAPDH-R	5’-CCCTTTTGGCTCCACCCTTC-3’
H-GAPDH-F	5’-TGGGTGTGAACCATGAGAAGT-3’
H-GAPDH-F	5’-AAGGCCATGCCAGTGAGCTT-3’
C-β-Actin-F	5’-GCCAACAGAGAGAAGATGACAC-3’
C-β-Actin-R	5’-GTAACACCATCACCAGAGTCCA-3’
P-β-Actin-F	5’-GACCTGACCGACTACCTCAT-3’
P-β-Actin-R	5’-CGTAGAGGTCCTTCCTGATGT-3’
M-circSOCS7-F	5’-TGGTACTGGGGACCTATGAAT-3’
M-circSOCS7-R	5’-GGATTCTGACGCTCTGATGG-3’
M-circIRF2-F	5’-ATGTGCCATGAATTCCCTGC-3’
M-circIRF2-R	5’-TCCTTTTCCACGTCCCATCC-3’
M-circTrappc9-F	5’-AGCTCATCCTGGAACACCTG-3’
M-circTrappc9-R	5’-TCTCTCTTCTTCCAGCTGCT-3’
IAV-NP-F	5’-TCAAACGTGGGATCAATG-3’
IAV-NP-R	5’-GTGCAGACCGTGCTAGAA-3’
IAV-NS1-F	5’-ATGTTATTTGCTCAAAACTATTC-3’
IAV-NS1-R	5’-CTACCTAACTGACATGACTCTT-3’
PRV-gE-F	5’-GCCGAGTACGTCACGGTCATCAAG-3’
PRV-gE-R	5’-GAGCACAGCACGCAGAGCCAGA-3’
H9N2-NP-F	5’-CCTGCTTGTGTGTACGGACT-3’
H9N2-NP-R	5’-TGAAGCAGACGGAAAGGGTC-3’
SeV-NP-F	5’- ATAAGTCGGGAGGAGGTGCT-3’
SeV-NP-R	5’- GTTGACCCTGGAAGAGTGGG-3’

MDRV-P10.8-F	5’-ATGGCTGACGCTTTTGAAGT-3’
MDRV-P10.8-R	5’-TAGTTAGATCTCGAGAGCCCG-3’
M-MerTK-F	5’-ACGTTGGTGGATACGTGCAT-3’
M-MerTK-R	5’-CTCTTCCCACTTCTCGGCAG-3’
H-MerTK-F	5’-TCGCTTCCTTCAGCATAACCA-3’
H-MerTK-R	5’-CAGGCCTGGAACAGTTAGCA-3’
M-circMerTK-F	5’-TGTCACCAGAAACACAGCCT-3’
M-circMerTK-R	5’-GGAGTCCTTGAACTTCCACG-3’
M-circMerTK-qF	5’-CCTCCTGAGCCCGTCAATATC-3’
M-circMerTK-qR	5’-TGAACTTCCACGTATATGGGATCA-3’
H-circMerTK-F	5’-AGCCCGTCAACATTTTCTGG-3’
H-circMerTK-R	5’-TCATGCTCTCAGGCTGCTTA-3’
H-circMerTK-qF	5’-GTGCTAACTGTTCCAGCATAACC-3’
H-circMerTK-qR	5’-CATGCTCTCAGGCTGCTTAGT-3’
C- circMerTK-F	5’-TTCTGACTGTCCCAGCATCC-3’
C- circMerTK-R	5’-GGCTGTTGAATGAAGTGTGGG-3’
P-circMerTK-F	5’-TACGAGGCAGCCTGAGAGTAT-3’
P-circMerTK-R	5’-GCACACTGGTTATGCAGGAA-3’
M-Mx1-F	5’-AGTAAGTGCAGCTGGTTCCT-3’
M-Mx1-R	5’-AAGGATCATTGCACAGCGAC-3’
H-Mx1-F	5’-GACATTCGGCTGTTTACC-3’
H-Mx1-R	5’-GCGGTTCTGTGGAGGTTA-3’
M-IFN-β-F	5’-GGTCCGAGCAGAGATCTTCA-3’
M-IFN-β-R	5’-CACTACCAGTCCCAGAGTCC-3’
H-IFN-β-F	5’-ACCAACAAGTGTCTCCTCCAA-3’
H-IFN-β-R	5’-TCTCCTCAGGGATGTCAAAGT-3’
H-IFITM3-F	5’-TGGCCAGCCCCCCAACTAT-3’
H-IFITM3-R	5’-CATAGGCCTGGAAGATCAG-3’

H-ISG15-F	5’- AGCATCTTCACCGTCAGGTC-3’
H-ISG15-R	5’- GCGAACTCATCTTTGCCAGT-3’
	shRNA sequence
Human sh-circMerTK	5’- AACTGTTCCAGCATAACCAGT-3’
Human sh-MerTK	5’- GATAATATATATTTATTTAAA-3’
Mouse sh-circMerTK	5’- CGTACCTGCATAGCCAGTG-3’
Human sh-IRF3	5’- CATTGTAGATCTGATTACCTTC-3’
Human sh-IRF7	5’- GCCTCTATGACGACATCGAGT-3’
Human sh-MDA5	5’- CCAACAAAGAAGCAGTGTATA-3’
Human sh-MAVS	5’- GGCAGGTCAGTTAACAATTTA-3’
	sgRNA sequence
Human sgRNA: IFNAR1 #1	5’-CTGCGGCGGCTCCCAGATGA-3’
Human sgRNA: IFNAR1 #2	5’-CCAGATGATGGTCGTCCTCC-3’
Human sgRNA: RIG-I #1	5’-ACGCAGCCTGCAAGCCTTCC-3’
Human sgRNA: RIG-I #2	5’-AAGCCTTCCAGGATTATATC-3’

Supplementary figure legends

FIG S1 (A) Schematic illustration of the circMerTK structure and the design strategy for the divergent primers (circ-F, circ-R) to target only the circular RNA and not the linear mRNA or pre-MerTK. (B) The schematic drawing represents the mouse homolog, and (C) the human homolog of circMerTK formed by backsplicing between exon 3 and exon 4 of MERTK pre-mRNA. The top part is the Sanger sequencing data; the arrow points at the backsplicing junction site. Schematic representation was generated by circPrimer2.0. (D) A multiple sequence alignment of human circMerTK (hsa-circMerTK) and the mouse circMerTK homolog (mmu-circMerTK), was made using basic local alignment search tool (BLAST, https://blast.ncbi.nlm.nih.gov/Blast.cgi).

FIG S2 (A) Wild type (WT) 293T and RIG-I knockout (KO) 293T cell lines were examined for RIG-I protein expression by western blot. (B) MLE-12, and (C) 293T cell lines were transfected with poly(I:C) at indicated concentrations for 4 h. Then, the levels of circMerTK and MX1 were analyzed by RT-PCR. (D) A549 cells were either treated with BAY 11-7082 or DMSO and then infected with IAV PR8 or mock-infected for 14 hours. RT-PCR assessed the expression of circMerTK. Shown are representative data from three independent experiments. IAV-NP: IAV nucleoprotein; WB: western blot.

FIG S3 (A) Schematic illustration of the shRNA designed to target the human circMerTK without altering the expression of linear MerTK. (B) The transduction efficiency of NIH/3T3 cells stably expressing pSIH-H1-GFP vectors targeting circMerTK or luciferase control, was measured by visualizing the green fluorescent protein (GFP) fluorescence activity. (C-D) The expression levels of circMerTK and MerTK mRNA were assessed in sh-luc and sh-circMerTK NIH/3T3 cell lines by RT-qPCR. (E-F) The expression levels of circMerTK and MerTK mRNA were relatively quantified in sh-luc and sh-circMerTK A549 cell lines by RT-qPCR. (G) RT-qPCR was used to determine the mRNA levels of Sev-NP after SeV infection in sh-luc, and sh-circMerTK A549 cell lines for 16 hours (MOI = 0.5). (H) SeV titers in supernatants of SeV-infected sh-luc and sh-circMerTK A549 knockdown cells were measured at indicated time points by HA assay (MOI = 0.5). (I-J) The expression

levels of circMerTK and MerTK mRNA were assessed in sh-luc and sh-MerTK A549 cell lines by RT-PCR and RT-qPCR. Results are shown as representative of three independent experiments with similar results. Data represent the mean values ± SD (n=3; ns= not significant, ***p* < 0.01). IAV-NS1: IAV non-structural protein 1; IAV-NP: IAV nucleoprotein; SeV-NP: SeV nucleocapsid protein; WB: western blot.

FIG S4 (A-B) The circMerTK and MerTK mRNA expression levels were assessed in pLC5-EV and pLC5-circMerTK NIH/3T3 cell lines by RT-qPCR. (C-D) The circMerTK and MerTK mRNA expression levels were evaluated in pLC5-EV and pLC5-circMerTK A549 cell lines by RT-qPCR. (E) RT-qPCR was used to determine the mRNA levels of IAV-NP after IAV PR8 infection in pLC5-EV, and pLC5-circMerTK stably expressed A549 cell lines for 16 hours (MOI = 0.5). (F) Western blotting was utilized to determine the protein levels of IAV-NP and IAV-NS1 after IAV PR8 infection of 293T stably expressing pLC5-EV or pLC5-circMerTK. (G) RT-qPCR was used to determine the mRNA levels of Sev-NP after SeV infection in pLC5-EV, and pLC5-circMerTK A549 cell lines for 16 hours (MOI = 0.5). (H) SeV titers in supernatants of SeV-infected pLC5-EV and pLC5-circMerTK A549 overexpression cells were measured at indicated time points by HA assay (MOI = 0.5). Results are shown as representative of three independent experiments with similar results. Data represent the mean values ± SD (n=3; ns= not significant, **p < 0.01,***p < 0.001). IAV-NS1: IAV non-structural protein 1; IAV-NP: IAV nucleoprotein; SeV-NP: SeV nucleocapsid protein; WB: western blot.

FIG S5 (A) A549 cell lines stably expressing specific shRNAs targeting circMerTK or luciferase were infected with or without IAV PR8 for 14 h. IFN-β mRNA expression levels were relatively quantified by RT-qPCR. (B) A549 cell lines stably expressing pLC5-EV or pLC5-circMerTK were challenged with or without IAV PR8 for 14 h. IFN-β mRNA expression levels were relatively quantified by RT-qPCR. Data represent mean ± SD (n=3; **p < 0.01).

FIG S6 (A) A549 cell lines were transfected with either negative control (NC) or miR-125a-3p inhibitor for 24 h, followed by challenging both cell lines with IAV PR8 infection for 14h. The expression levels of circMerTK were determined by RT-qPCR in challenged and unchallenged cell lines. (B) A549 cell lines were transfected with

either negative control (NC) or miR-125a-3p mimic for 24 h, followed by challenging both cell lines with IAV PR8 infection for 14h. The expression levels of circMerTK were determined by RT-qPCR in challenged and unchallenged cell lines. Data represent mean ± SD (n=3; **$p < 0.01$).
